# ^1^H NMR Study of the In Vitro Digestion of Highly Oxidized Soybean Oil and the Effect of the Presence of Ovalbumin

**DOI:** 10.3390/foods10071573

**Published:** 2021-07-06

**Authors:** Ana S. Martin-Rubio, Patricia Sopelana, María L. Ibargoitia, María D. Guillén

**Affiliations:** Food Technology, Faculty of Pharmacy, Lascaray Research Center, University of the Basque Country (UPV/EHU), 01006 Vitoria, Spain; anamaria.sanmartin@ehu.eus (A.S.M.-R.); patricia.sopelana@ehu.eus (P.S.); marialuisa.ibargoitia@ehu.eus (M.L.I.)

**Keywords:** oxidized soybean oil, in vitro digestion, ^1^H NMR, lipolysis, oxidation, ovalbumin, hydroperoxides, epoxides, aldehydes, keto-dienes, hydroxy-derivatives, furan-derivatives

## Abstract

Oxidized lipids containing a wide variety of potentially toxic compounds can be ingested through diet. However, their transformations during digestion are little known, despite this knowledge being essential in understanding their impact on human health. Considering this, the in vitro digestion process of highly oxidized soybean oil, containing compounds bearing hydroperoxy, aldehyde, epoxy, keto and hydroxy groups, among others, is studied by ^1^H nuclear magnetic resonance. Lipolysis extent, oxidation occurrence and the fate of oxidation products both present in the undigested oil and formed during digestion are analyzed. Furthermore, the effect during digestion of two different ovalbumin proportions on all the aforementioned issues is also addressed. It is proved that polyunsaturated group bioaccessibility is affected by both a decrease in lipolysis and oxidation occurrence during digestion. While hydroperoxide level declines throughout this process, epoxy-compounds, keto-dienes, hydroxy-compounds, furan-derivatives and n-alkanals persist to a great extent or even increase. Conversely, α,β-unsaturated aldehydes, especially the very reactive and toxic oxygenated ones, diminish, although part of them remains in the digestates. While a low ovalbumin proportion hardly affects oil evolution during digestion, at a high level it diminishes oxidation and reduces the concentration of potentially bioaccessible toxic oxidation compounds.

## 1. Introduction

Food lipids can undergo oxidative reactions during their processing and storage, which adversely affect food quality and safety [1]. The nature and amount of the compounds generated during oxidation will vary depending on different factors such as lipid composition, temperature, time or aeration [2]. Therefore, different types of lipid oxidation products can be ingested through diet, either coming from high temperature treatments such as frying [3] or generated under other conditions. Indeed, oxidation compounds can also be present in oils and fats not submitted to high temperatures [4], possibly due to their generation during manufacturing stages and/or storage.

The presence of oxidation products in dietary lipids makes it an important matter for several reasons, such as those relating to the lipid digestion process, which constitutes the prelude to the effect of this kind of food components on the body. First of all, several in vitro and in vivo studies have revealed that the thermoxidation of lipids and the presence in them of triglyceride dimers and polymers negatively affects lipid digestibility by reducing their lipolysis extent and/or absorption [5,6,7]. These works, which have provided very valuable knowledge about the digestibility of thermoxidized lipids, have been conducted using thermodegraded oils coming from processes at frying temperature. However, very little is known about the digestion of lipids that have undergone oxidation at lower, moderate temperatures which, as above-mentioned, will present a different profile of oxidation products. In this regard, previous studies by our research group, carried out with slightly oxidized polyunsaturated oils, containing basically hydroperoxides (primary oxidation products), have shown that the level of lipolysis reached after submission to in vitro gastrointestinal digestion is lower than that found when digesting these same unoxidized oils [8,9,10].

In addition to lipolysis, which is the main reaction affecting lipids during digestion, other reactions can also take place, among which oxidation can be cited due to its repercussions on human health [11,12]. It must be noticed that the occurrence of oxidation during digestion not only can lead to the degradation of main and minor oil components [8,10,13], with the subsequent nutritional value losses, but also to the generation of toxic oxidation products [14,15,16,17]. Concerning this issue, it is worth pointing out that several variables can affect oxidation occurrence during digestion, among which the initial oxidative status of the lipids submitted to digestion can be mentioned [12].

The third important issue that should be taken into account when analyzing the digestion process of oxidized lipids is the evolution or fate of the oxidation products that they contain, since many of them are well-known reactive and toxic compounds. This is the case of oxygenated α,β-unsaturated aldehydes [18], certain monoepoxides coming from different fatty acyl groups [19,20], keto-epoxy-monoene-derivatives [21,22] or certain dihydroxy-derivatives coming from linoleic groups [23], among others. All of them have been proved recently to be generated during the oxidation of an edible oil under accelerated storage conditions [24], so they could also be present in dietary oxidized lipids. Therefore, depending on their evolution during digestion, oxidation products could react with different biological components of the gastrointestinal tract, especially of the mucosa [25], and also be absorbed, thus reaching different targets. Actually, the in vivo and/or in vitro absorption of hydroperoxy-, hydroxy-, epoxy- and keto-fatty acids, as well as of 4-hydroxy-(*E*)-2-alkenals has already been described [26,27,28,29,30]. Notwithstanding, in spite of the relevance of this topic, very little is known about the evolution of oxidation products during digestion. In addition, in some cases dissenting results can be found, for example those regarding the persistence of hydroperoxides in the gastrointestinal tract [31,32]. With respect to secondary oxidation compounds, only a few studies have been conducted to assess the fate during digestion of aldehydes [33,34,35] and of some epoxides [29,36], as far as we know. 

With all the above in mind, the in vitro digestion process of highly oxidized virgin and refined soybean oils, containing a wide variety of oxidation products, will be addressed in this work. Lipolysis extent, the occurrence of oxidation during digestion and the fate of oxidation products, either present in the undigested oxidized oils or formed during the in vitro digestion process, will be examined. These include compounds bearing hydroperoxy, aldehyde, epoxy, keto and hydroxy groups, among others. It must be remarked that, as far as we know, the study of the simultaneous evolution under in vitro digestion conditions of a so broad range of lipid oxidation products, some of them considered for the first time in this type of studies, has not been tackled before.

Furthermore, taking into account that the presence of other nutrients such as proteins can affect lipolysis extent and/or the oxidation progress during in vitro digestion [9,37,38], the effect of two different proportions of ovalbumin during the in vitro digestion of the above-mentioned oxidized oils will also be addressed. This latter study is considered of interest because previous findings of our research group revealed that the addition of ovalbumin can considerably improve lipolysis extent during the in vitro digestion of slightly oxidized soybean oil [9]. However, the effect of the presence of this protein on the reactions taking place during the in vitro digestion of this oil in a much more advanced oxidation stage is not yet known.

The technique employed to accomplish the goals of this work was ^1^H NMR, which permits one to study both lipolysis degree and the advance and/or occurrence of oxidation during the digestion process in a global way, as well as to analyze the fate of oxidation products.

The study of the evolution of oxidized lipids and their oxidation products during digestion, and of the parameters influencing this evolution, can be considered an issue of primary interest in furthering the intricate task of assessing how dietary lipids can affect human health. Indeed, the health hazards posed by oxidized lipids are of great concern nowadays [39]. In this context, the results of this work are expected to provide very valuable knowledge on several subjects, namely: (i) the degree of lipolysis and oxidation during the in vitro digestion of oxidized oils; (ii) the extent to which the various classes of oxidation products remain after digestion; (iii) how other food components such as ovalbumin can affect this evolution.

## 2. Materials and Methods 

### 2.1. Samples Subject of Study

The samples subject of study were two commercial soybean oils, one virgin and the other refined, both in an advanced degree of oxidation, containing primary and a wide variety of secondary oxidation products. In order to obtain these samples, 10 g of each of the two oils were weighed in glass Petri dishes of 80 mm diameter and submitted to an accelerated storage process at 70 °C in a convection oven with aeration (Memmert GmbH+Co, Schwabach, Germany) for 8 and 9 days, respectively, in order to obtain samples with similar oxidation degrees; these were designated as VSX (oxidized virgin soybean oil) and RSX (oxidized refined soybean oil).

In addition, samples were prepared by mixing food grade ovalbumin, acquired from a protein supplier (Apasa SA, Astigarraga, Spain), with each one of the highly oxidized oils just before digestion. Two different proportions of ovalbumin were tested: 0.26 g per g of oil (low level of ovalbumin, 26% in weight: LO) and 2.6 g per g of oil (high ovalbumin proportion, 260% in weight: HO). The samples derived from the virgin oil were designated as VSXLO and VSXHO, and those prepared from the refined oil RSXLO and RSXHO. The amounts of oil and protein in samples VSXHO and RSXHO were selected trying to simulate the proportions of fat and protein found in a food system where protein is in a much higher proportion than lipids, as in a previous study [37]. Conversely, in VSXLO and RSXLO samples, the proportion of protein was considerably lower than that of lipids.

### 2.2. In Vitro Gastrointestinal Digestion

All the samples mentioned above (0.5 g of oil in all cases) were digested following an in vitro digestion protocol described in previous works [8,9,10]. This in vitro digestion model was developed on a physiological basis and validated by Versantvoort and coworkers [40,41], and was further slightly modified to achieve a lipid digestion level similar to that obtained in vivo [42]. This involves three steps to simulate digestive processes in the mouth, stomach and small intestine. The composition of the corresponding digestive juices is given in the Supplementary Material (see Table S1), together with other details of the in vitro digestion process. All the reagents used were acquired from Sigma-Aldrich (St. Louis, MO, USA).

Two digestion experiments, each including duplicate samples of all the oil systems studied, were performed. Blank samples corresponding to the juices submitted to digestive conditions were also taken for further analysis.

### 2.3. Lipid Extraction of the Digestates

Lipids of the digestates were extracted using dichloromethane as solvent (HPLC grade, Scharlab, Barcelona, Spain) and with the aid of a Sigma 3K30 centrifugal machine working at 10,000 rpm (Sigma Laboratory Centrifuges, Germany), following the same methodology as in a previous study [9]. The procedure involves three liquid–liquid extraction stages with 20 mL of dichloromethane each. Afterwards, to ensure a complete protonation of fatty acids and/or the dissociation of the potential salts formed, the remaining water phase was acidified to pH 2 with HCl (37%) and a second extraction was carried out, also in three steps. All these extracts were collected together and, after removing the solvent, the concentrated lipid residue was stored at −80 °C until its analysis.

In order to verify that the methodology used was suitable to efficiently extract the different types of oxidation products present in the undigested oils, samples of the starting oxidized oils were mixed with the digestive juices after being submitted to the digestion process, and this mixture was extracted following the same procedure mentioned above, complete recoveries being achieved in all cases.

### 2.4. Analysis by ^1^H NMR

#### 2.4.1. Operating Conditions

The ^1^H NMR spectra of the starting oxidized oils (VSX and RSX) and of the lipid extracts of the oil samples digested without ovalbumin (DVSX and DRSX), with a low proportion (26% in weight) of ovalbumin (DVSXLO and DRSXLO) and with a high proportion (260% in weight) of this protein (DVSXHO and DRSXHO) were acquired in quadruplicate using a Bruker Avance 400 spectrometer (Bruker, Rheinstetten, Germany) operating at 400 MHz. For this purpose, approximately 0.16 g of the above-mentioned oils or lipid extracts were dissolved in 400 µL of deuterated chloroform that contained tetramethylsilane (TMS) as internal reference (Cortec, Paris, France).

In order to select the most appropriate values to obtain accurate quantitative results in the smallest possible period of time, a broad range of recycling times and relaxation delays were tested in the acquisition of the ^1^H NMR spectra. In this way, the acquisition parameters finally used were: spectral width 6410 Hz, relaxation delay 3 s, number of scans 64, acquisition time 4.819 s and pulse width 90°, in agreement with previous studies on edible oils and fats carried out in our laboratory [24,38,43,44,45,46,47,48,49,50,51]. The relaxation delay and acquisition time used allow the complete relaxation of the protons, the signal areas thus being proportional to the number of protons that generate them, being possible their use for quantitative purposes. The experiments were carried out at 25 °C.

#### 2.4.2. Identification of Some Compounds and Structures

**Figure 1:**
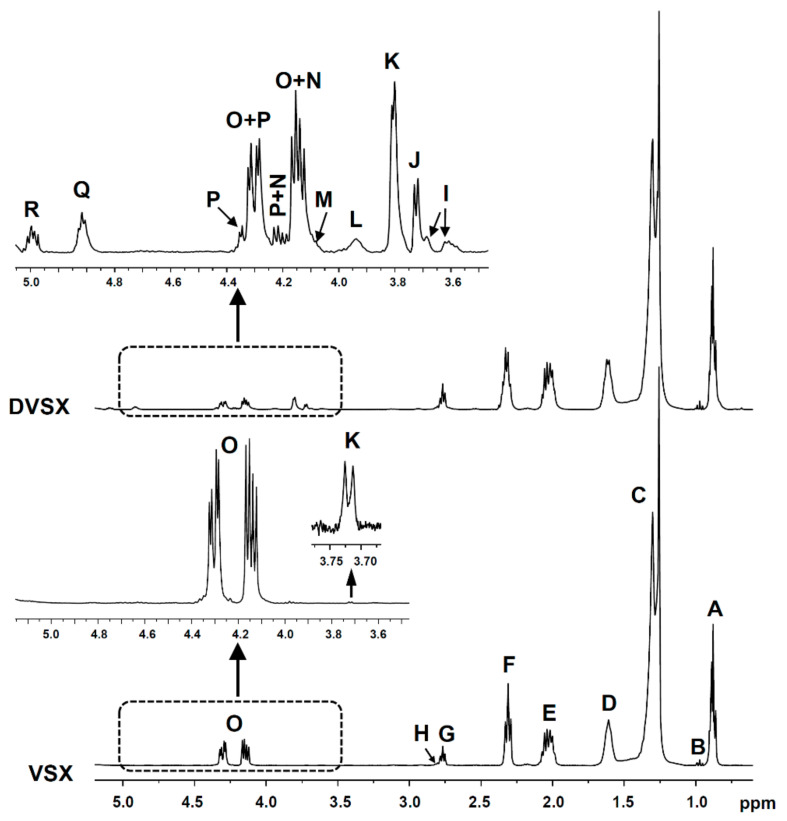
Region between 0.5 and 5.1 ppm of the ^1^H NMR spectra of VSX and DVSX samples, and enlargements of the region between 3.5 and 5.1 ppm, where the main proton signals of glycerides appear. The signal letters agree with those of Tables S2 and S3 of the Supplementary Materials.

**Figure 2:**
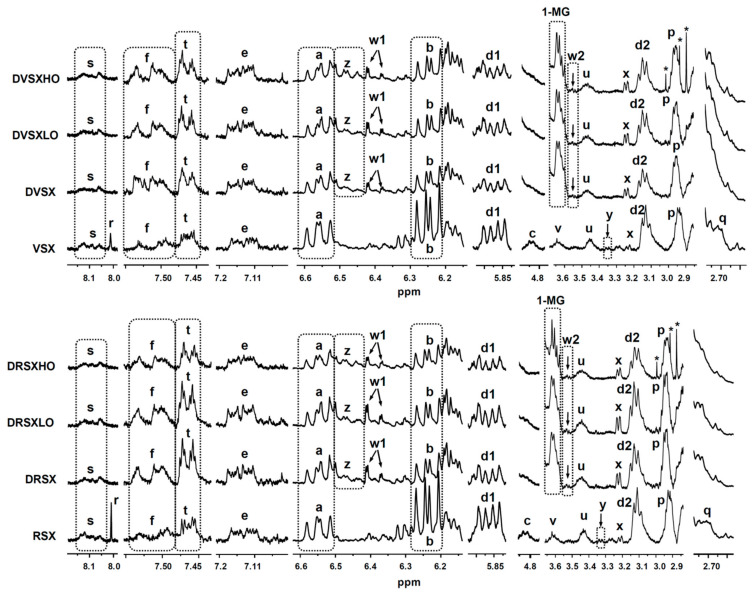
Enlargements of some regions of the ^1^H NMR spectra of the highly oxidized virgin and refined soybean oils (VSX and RSX), and of the lipid extracts obtained after their in vitro digestion in the absence of ovalbumin (DVSX and DRSX), as well as in the presence of a low (DVSXLO and DRSXLO) and a high proportion of ovalbumin (DVSXHO and DRSXHO). The signal letters agree with those in Tables S4, S5, S7, S8 and S9. Signals marked with an asterisk are considered to come from the ovalbumin sample used. The plots corresponding to the same ^1^H NMR spectral region are presented at a fixed value of absolute intensity, for them to be valid for comparative purposes. 1-MG: 1-monoglycerides.

**Figure 3:**
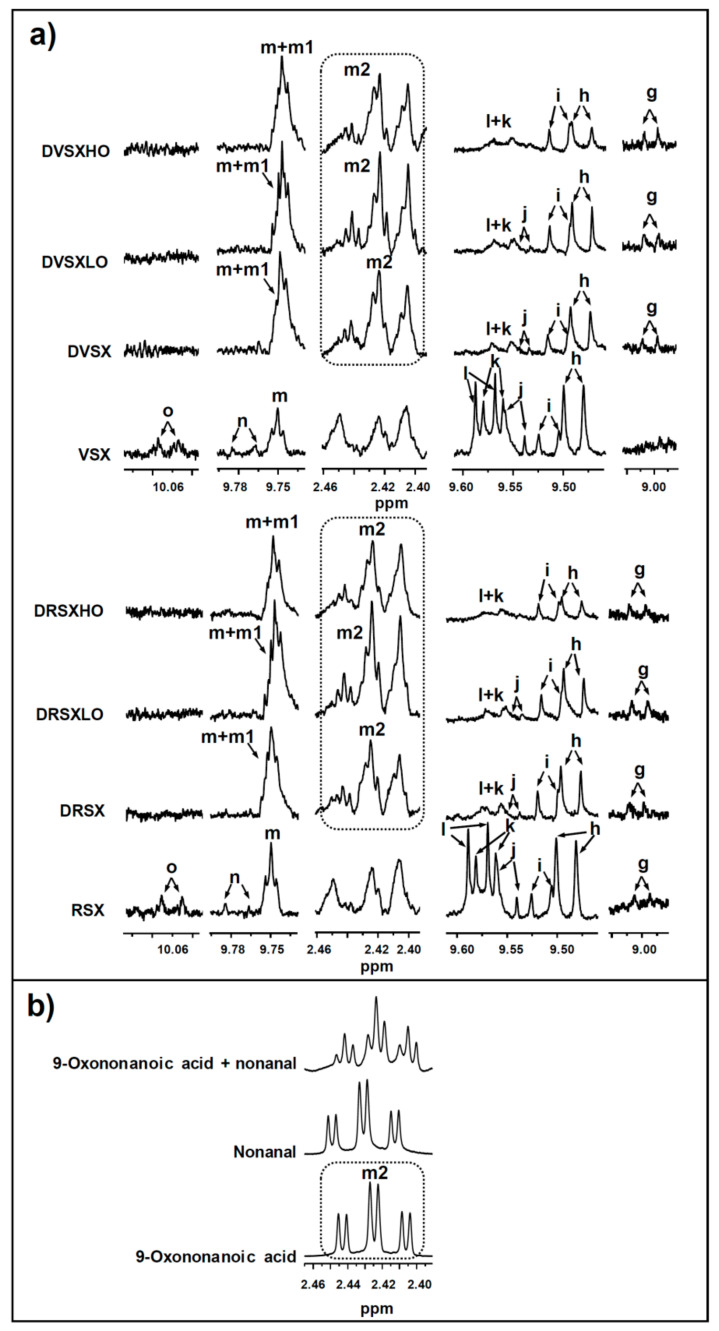
Enlargements of: (**a**) some regions of aldehydic signals of the ^1^H NMR spectra of the highly oxidized virgin and refined soybean oils (VSX and RSX), and of the lipid extracts obtained after their in vitro digestion in the absence of ovalbumin (DVSX and DRSX), as well as in the presence of a low (DVSXLO and DRSXLO) and a high proportion of ovalbumin (DVSXHO and DRSXHO); and (**b**) one of the ^1^H NMR spectral regions where typical signals of 9-oxononanoic acid and of saturated aldehydes appear, corresponding to pure 9-oxononanoic acid, to nonanal and to a mixture of both. The signal letters agree with those in Table S6. The plots corresponding to the same ^1^H NMR spectral region are presented at a fixed value of absolute intensity, for them to be valid for comparative purposes.

The chemical shift assignments and multiplicities of the ^1^H NMR signals in CDCl_3_ of the main protons of glycerides and of fatty acids and fatty acyl groups are displayed in Tables S2 and S3 (see Supplementary Material). The identification of the different types of oxidation products present in the oxidized oils subject of study and in the lipid extracts of the digestates obtained from them was carried out on the basis of the proton signal assignments shown in Tables S4–S9, made from bibliographic data and with the aid of several standard compounds, also given in the Supplementary Material. The intensity of some of the signals shown in these tables were used to quantify the concentration of the different kinds of above-mentioned structures in the several samples, as will be explained below. All these ^1^H NMR signals are named in the above-mentioned tables with the same letters as in Figure 1, Figure 2 and Figure 3a.

#### 2.4.3. Quantification from ^1^H NMR Spectral Data

Bearing in mind that with the used acquisition parameters, mentioned above, the area of each ^1^H NMR spectral signal is proportional to the number of protons that generate it, and that the proportionality constant is the same for all kinds of protons, the area of some spectral signals was used to carry out different quantifications.

Among these is the estimation of the molar percentage of the different kinds of glyceryl structures in relation to the total glyceryl structures in a lipid sample. This estimation allows to know the lipolysis degree of the lipids of a sample and these data are essential in lipids digestion studies. These important compositional parameters can be estimated from the intensity of some specific ^1^H NMR signals, shown in Figure 1 and given in Tables S2 and S3, by using Equations (S1)–(S10), all of them in the Supplementary Materials. In this way, the molar percentages of triglycerides (TG%), 1,2-diglycerides (1,2-DG%), 1,3-diglycerides (1,3-DG%), 2-monoglycerides (2-MG%), 1-monoglycerides (1-MG%) and glycerol (Gol%), in relation to the total number of glyceryl structures present in the various lipid samples, can be estimated. Although glycerol is formed during digestion, due to its polar nature, it is not present in the lipid extract of the digestates. However, its concentration can be determined indirectly. This is possible because the concentration of total fatty acids plus acyl groups, of only acyl groups, and of fatty acids liberated in the formation of diglycerides and monoglycerides can be determined from ^1^H NMR data. This method was validated in a previous study carried out with different mixtures made with several standard compounds having different kinds of glyceryl structures [48].

Another determination able to be made from the intensity of some ^1^H NMR spectral signals of lipid samples shown in Figure 1 and in Table S3 is the molar concentration of the different kinds of fatty acyl groups plus fatty acids contained in them. To this aim, several sound methods, most of them expressing the concentration of the different kinds of acyl groups in molar percentage, have been published [38,44,45,49,52], and some of them was even validated by using mixtures of different standard compounds [44]. In this study the molar concentrations of linolenic acyl groups plus linolenic fatty acids on the one hand, and of linoleic acyl groups plus linoleic fatty acids on the other have been determined in relation to the molar concentration of total fatty acyl groups plus fatty acids (AG + FA) in the lipid samples. This determination in the oil samples before digestion and in their corresponding digestates allows to know whether the digestion has provoked changes in the concentration of these structures that evidence their degradation during this process. This determination was carried out by using Equations (S11) and (S12) and the intensity of some ^1^H NMR signals indicated in Table S3 (see Supplementary Materials) giving the concentrations of the above-mentioned structures in millimoles per mole of total AG + FA (mmol/mol AG + FA).

All the structures mentioned above are main structures in lipids. However, lipid samples can also contain minor components, either natural or formed in lipid degradation processes. If these are in enough concentration to be detected by ^1^H NMR, their concentration in the sample can also be determined, provided that some of their signals do not overlap with other ones [24,47]. This determination can be made either by adding a standard compound or by using certain compounds or structures present in the sample as reference. In the case of complex mixtures, such as those of lipids, it is not easy to find standard compounds whose ^1^H NMR signals do not overlap with others of interest for the study. In previous studies we have taken as standard reference the non-deuterated chloroform that always accompanies the deuterated chloroform [47,49], but its high volatility is a great inconvenient for the repeatability of the experiments. For this reason, references such as either triglycerides [24,51] or the total fatty acids (FA) plus fatty acyl groups (AG) [15,38] present in the sample have been used to quantify minor components in mmol in relation to these references given in mol. In this study, the determination of the concentration of minor components in the samples before digestion and in their digestates, was made by using the total concentration of fatty acyl groups plus fatty acids as reference. In this way, the concentrations of minor components were determined by introducing the area of some of the spectral signals shown in Figure 1, Figure 2 and Figure 3a and in Tables from S3 to S9 in Equation (S13) of the Supplementary Material. This determination in the cited samples allows one to go in depth on the fate during digestion of minor components of the sample submitted to this process, as well as to know whether new components are formed in them.

### 2.5. Statistical Analysis 

The significance of the differences on the several determinations made among the samples was determined by one-way variance analysis (ANOVA) followed by Tukey b test at *p* < 0.05, using SPSS Statistics 24 software (IBM, Armonk, NY, USA).

## 3. Results and Discussion

The results here presented address the study of: (i) the composition of the oil samples submitted to in vitro digestion; (ii) the lipolysis extent reached during digestion; (iii) the degradation level of oil acyl groups undergone during in vitro digestion; (iv) the fate of both the oxidation products present in the samples submitted to digestion and those generated during this process. Furthermore, the effect of the presence of variable proportions of ovalbumin on the above-mentioned issues (lipolysis, oxidation occurrence during digestion and fate of oxidation products) is also discussed.

### 3.1. Composition of the Oil Samples Subject of Study

As mentioned before, the samples submitted to digestion are virgin and refined soybean oils in an advanced oxidation stage (VSX and RSX). As Table 1 shows, both oxidized oils maintain the same glyceride composition as that of these oils before oxidation [53], because during the accelerated storage process no hydrolysis takes place. However, some of these glycerides, mainly triglycerides (TG) and a very small percentage of diglycerides (DG), can be present as monomers, dimers, oligomers or polymers. These di-, oligo- or polymerized structures cannot be discarded due to the high oxidation degree of the oil samples subject of study.

Furthermore, as is common in soybean oils, fatty acyl groups such as linolenic (Ln) and linoleic (L) are present in these oxidized oils. Nevertheless, due to the degradation undergone during the accelerated storage process, their concentrations, given in Table 2 and estimated by using, as mentioned above, the area of the signals indicated in Table S3, are considerably lower than those of the same unoxidized oils [8]. 

As is usual, the degradation of these acyl groups during accelerated storage has as a consequence the formation of a pleiad of oxidation derivatives, which are present in VSX and RSX samples, as Table 3 shows. 

In agreement with previous findings [53], both oxidized oils still contain significant concentrations of monohydroperoxides supported on acyl chains having (*Z*,*E*)- and (*E*,*E*)-conjugated dienes (signals “a” and “b”, respectively, in Figure 2 and Table S4, and estimated concentrations in Table 3). Moreover, several kinds of secondary oxidation products are also present, which include dihydroperoxide non-conjugated diene-derivatives (signal “c” in Figure 2, signal assignment in Table S4 and estimated concentrations in Table 3), and hydroperoxy-epoxy-(*E*)-monoenes, these latter in important concentration (signals “d1” and “d2” in Figure 2 and Table S4, and estimated concentrations in Table 3).

In addition, keto-(*Z*,*E*)- and -(*E*,*E*)-conjugated dienes (signals “f” and “e” in Figure 2, signal assignments in Table S5 and estimated concentrations in Table 3) and several classes of aldehydes are present in these oil samples, including the very reactive oxygenated α,β-unsaturated ones such as 4-hydroperoxy-, 4-hydroxy-, 4,5-epoxy- and 4-oxo-(*E*)-2-alkenals, and also (*E*)-2-alkenals, n-alkanals, (*E*,*E*)-2,4-alkadienals, (*Z*)-2-alkenals and 2,3-epoxy-alkanals, these latter only detectable in RSX sample (signals “l”, “k”, “j”, “n”, “h”, “m”, “i”, “o” and “g”, respectively, in Figure 3a). The assignments of the various aldehydic signals are given in Table S6 and their estimated concentrations in Table 3. 

Furthermore, (*Z*)- and (*E*)-monoepoxides with only an epoxy group were also tentatively identified in the oxidized oils submitted to digestion (signals “p” and “q”, respectively, in Figure 2 and signal assignments in Table S7), as well as formic acid and structures having formate group (signals “r” and “s”, respectively, in Figure 2 and signal assignments in Table S7). In addition, structures supporting 5-alkyl-(5H)-furan-2-one groups (signal “t” in Figure 2 and signal assignment in Table S7), are also present in VSX and RSX oils. The estimated concentrations of all these types of oxidation derivatives are given in Table 3. 

Moreover, other types of structures tentatively bearing either hydroxy or ether functional groups were also found in these oxidized oil samples (signals “u” and “v” in Figure 2, signal assignments in Table S7 and estimated concentrations in Table 3); the first could be due to dihydroxy-derivatives such as leukotoxin and/or isoleukotoxin diols, and/or to other kinds of hydroxy-derivatives, and the second ones could be due to protons of ether groups of dimeric structures formed between acyl groups of the same or different molecule. 

Given the advanced oxidation state of the oils involved in this study, two different groups of keto-epoxy-(*E*)-monoenes, exhibiting either non-vicinal or vicinal keto and epoxy groups were detected. The assignments of their signals are given in Table S8. Within the group of non-vicinal keto-epoxy-(*E*)-monoenes, in turn, keto-(*E*)-epoxy- and keto-(*Z*)-epoxy-(*E*)-monoenes can be distinguished. As Table S8 shows, these two types of keto-epoxy-(*E*)-monoene-derivatives give a common signal at 3.20 ppm, which is observed in the ^1^H NMR spectra of VSX and RSX samples (see signal “x” in Figure 2). However, individual signals of each kind of these compounds cannot be clearly distinguished in the spectra of the undigested oils due to their overlapping with others, so their concentration has been estimated jointly from signal “x” (see Table 3). Regarding vicinal keto-(*E*)-epoxy-(*E*)-monoenes, the estimation of their concentration, shown in Table 3, was made by using the area of signal “y”, noticeable in the spectra of VSX and RSX samples (see Figure 2).

As has been commented above (see Section 2.1), in addition to the just described VSX and RSX oils, mixtures of these same samples with two different levels of ovalbumin: 26% in weight in VSXLO and RSXLO, and 260% in weight in VSXHO and RSXHO were also submitted to in vitro digestion.

### 3.2. Lipolysis Extent Reached in the In Vitro Digestion of the Several Samples and Consequences

#### 3.2.1. In the In Vitro Digestion of VSX and RSX Samples

**Figure 4:**
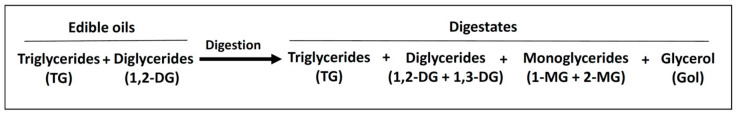
Usual lipolytic changes during digestion of edible oils.

The estimation of the extent of the main reaction occurring during the in vitro digestion of lipids, as mentioned before (see Section 2.4.3), was accomplished by comparison of the percentage of the different glyceride structures in the oil samples before digestion and in the digestates (see Figure 4), both being studied by means of ^1^H NMR.

Table 1 gives the molar percentages of triglycerides, TG, of the other glycerides (1,2-DG, 1,3-DG, 2-MG and 1-MG) and of glycerol, referred to the total glyceryl structures, in VSX and RSX samples before being submitted to digestion, and in their digestates DVSX and DRSX. It can be observed that the digestion process reduces the molar percentage of TG to near 35% in both digestates DVSX and DRSX, and the distribution of the different types of glycerides obtained matches well with that observed in previous works conducted with slightly oxidized oils [9,10]. 

From data in Table 1 it is evident that near 44.3%, and near 44.6% of the ester bonds present in VSX and RSX samples, respectively, were hydrolyzed during digestion. The lipolysis extent in the digestates of these oil samples, in terms of percentage of hydrolyzed glyceryl ester bonds, is lower than that found in the digestates of the same unoxidized oils [8] and only somewhat lower than that of the digestates of the same oils when slightly oxidized [9], where the proportions of hydrolyzed ester bonds were 48.4% and 46.0% for the virgin and the refined oils, respectively.

The smaller lipolysis extent reached during the digestion of these highly oxidized soybean oils (VSX and RSX) in relation to the same unoxidized or slightly oxidized oils can be due to potential inactivation of some lipolytic enzymes by reactions with some oil oxidation derivatives. Among these, the well-known reaction between amino groups of enzymes and carbonyl groups of certain oxidation compounds can be cited. In spite of the importance of this fact, as far as we know, there are only a few studies dealing with the impact of specific oxidation products on the activity of digestive enzymes [54,55,56,57]. Moreover, of these, only that carried out by Matsushita [55] includes pancreatic lipase, the main enzyme involved in food lipid hydrolysis. This researcher showed that while pancreatic lipase was hardly inhibited by linoleic acid secondary oxidation products after 20 min of incubation at 37 °C, linoleic acid hydroperoxides caused a higher lipase inhibition extent, although not proportional to their concentration. Moreover, it was shown that when hydroperoxides were supported on TG instead of fatty acids, the effect observed on enzyme activity was less pronounced, with only small differences between the inhibition degrees caused by concentrations of 1 and 10 μmol of TG hydroperoxides per mL of lipase solution. Furthermore, it is worth pointing out that, in the digestive juices used, as well as lipases, there are other enzymes and proteins such as bovine serum albumin (see Table S1), which could also react with the oxidation products present in the samples submitted to digestion, and maybe to a greater extent than pancreatic lipase.

In addition to the susceptibility of each enzyme to inactivation by specific oxidation products, another factor that could affect enzyme performance when digesting thermally degraded oils is the polarity of the system [58]. According to Arroyo, Sánchez-Muniz, Cuesta, Sinisterra and Sánchez-Montero [59], the polar compounds formed during thermal lipid degradation could act as surfactants favoring the formation of a microemulsion, and in turn, the action of pancreatic lipase. Therefore, all the aforementioned demonstrates that several factors may influence lipolysis extent.

It must be remarked that the lipolysis degree reached during digestion may be seen as a very important matter, since it affects the bioaccessibility of oil main components (fatty acyl groups supported on TG), and therefore their nutritional effect. In a general way, the in vitro bioaccessibility of a compound can be defined as the concentration of the compound that remains absorbable after in vitro digestion. For oil main components it can be expressed by the concentration of fatty acids and monoglycerides in the digestate referred to the total concentration of fatty acids and acyl groups, these latter representing all main components potentially absorbable before digestion. The bioaccessibility of oil main components thus estimated is near 53.3% in DVSX and near 54.1% in DRSX. However, it must be pointed out that the real bioaccessibility of the main components of these very oxidized oils could be smaller than seen in the above data, due to their possible content of dimeric, oligomeric or polymeric structures, formed by carbon–carbon bonds, ether bridges or peroxide linkages that cannot be broken by digestive lipases. Nevertheless, in view of a previous study on the digestibility of oxidized linoleic acid [60], it could be thought that only those dimerized or polymerized structures formed by carbon–carbon bonds between not modified acyl groups could be considered to have very low digestibility. Finally, it should be added that some of the absorbable fatty acids and monoglycerides could support oxidation derived functional groups, such as hydroperoxy, epoxy, hydroxy or oxo, which are potentially harmful for human health [19,61,62,63].

#### 3.2.2. In the In Vitro Digestion of VSX and RSX Samples with Ovalbumin Added

In the oxidized oil samples containing 26% in weight of ovalbumin, named VSXLO and RSXLO, the presence of protein hardly affects the lipolysis extent reached during digestion (around 43.8% of hydrolyzed glyceryl ester bonds in DVSXLO and 44.1% in DRSXLO) in relation to that achieved in the digestion of VSX and RSX samples (see Section 3.2.1). Moreover, the lipolysis pattern (see Table 1) is also fairly similar in the digestates of both kinds of samples, even though a slight tendency to increase the proportions of 2-MG and 1,2-DG, and to decrease those of TG and glycerol could be glimpsed in the digestates of the samples having a low concentration of ovalbumin. Due to the scarce differences in lipolysis extent among the above sets of samples, it is also to be expected that the bioaccessibility of their main components will be similar. In fact, this parameter is near 53.4% in DVSXLO and near 54.7% in DRSXLO. 

Likewise, the lipolysis degree reached in the in vitro digestion of the oxidized oil samples containing 260% in weight of ovalbumin, named VSXHO and RSXHO, is very similar to that of the rest of samples studied (45.4% in DVSXHO and 44.3% in DRSXHO). These findings contrast with the great increase in lipolysis extent observed in the presence of this same proportion of ovalbumin during the in vitro digestion of less oxidized soybean oil samples [9] (from approximately 48% to 66% of hydrolyzed ester bonds in the case of the virgin oil, and from 46% to 63% for the refined one). This difference can be attributed to the great degradation level of the oil samples here involved, which could reduce the efficiency of ovalbumin in improving lipolysis.

However, it is remarkable that although the lipolysis extent is similar in the several kinds of digested samples (without or with added ovalbumin), the lipolysis pattern in the digestion of VSXHO and RSXHO is somewhat different, which could confirm the trend, only glimpsed, in the lipolysis pattern of the digestates of VSXLO and RSXLO. Thus, as Table 1 shows, when ovalbumin is present in high concentration, significant increments in the proportions of 1,2-DG and especially of 2-MG, together with decreases in the proportions of TG, 1-MG and glycerol, against the values found in the rest of digestates, are clearly observed.

Notwithstanding, these small changes in the glyceride profile of DVSXHO and DRSXHO samples compared to the rest of digestates, do not entail important variations in the bioaccessibility of their main components, which reaches 56.8% and 55.1%, respectively. As mentioned, these values could also be considered overestimated, because these samples can have a certain polymerization degree through different kinds of linkages between fatty acyl groups.

### 3.3. Changes in the Concentration of the Most Unsaturated Fatty Acyl Groups of the Oil Occurred during the In Vitro Digestion and Consequences

As mentioned in the introduction section, in addition to lipolysis, another reaction that can also affect lipids during in vitro digestion is oxidation [11,12,64]. This will affect mainly the most unsaturated acyl groups, provoking their degradation, for which reason changes in their concentration during the in vitro digestion of the several samples is addressed below.

#### 3.3.1. In the In Vitro Digestion of VSX and RSX Samples

Given that polyunsaturated groups are the most prone to oxidation, their concentrations, expressed in mmol/mol AG + FA, were determined before and after the in vitro digestion process, and the corresponding values are displayed in Table 2 (see samples VSX, RSX, DVSX and DRSX). They reveal that digested samples have smaller concentrations of both polyunsaturated linoleic and linolenic AG + FA than the undigested samples, this difference being more pronounced for linolenic than for linoleic groups. These results contrast with the findings for these same soybean oils having a lower oxidation degree [9], since significant variations in their concentrations of polyunsaturated acyl groups were not observed with digestion. This shows that oxidation occurs to a greater extent the higher the initial oxidation state of the oil samples is, in agreement with the findings of other authors [65]. Furthermore, it should be remembered that, due to the greater trend towards oxidation of free fatty acids and monoglycerides than that of triglycerides or diglycerides, it could be expected that the formation of new oxidation compounds during digestion, would occur in the former rather than in the latter. This fact would have, as a result, increased bioaccessibility of potentially toxic compounds. Nevertheless, the contribution of these new oxidation products formed during digestion to the total oxidation derivatives present in the digestates can be considered small compared with that of the samples submitted to digestion.

#### 3.3.2. In the In Vitro Digestion of VSX and RSX Samples with Ovalbumin Added

The presence of a low ovalbumin proportion in the samples subjected to digestion (VSXLO and RSXLO) does not produce changes in the degradation of polyunsaturated acyl groups compared with that observed in the digestion of VSX and RSX (see Table 2), this being in agreement with previous results [9].

However, as Table 2 shows, the presence of a high proportion of ovalbumin clearly reduces the degradation of the most unsaturated acyl groups during the in vitro digestion (see samples DVSXHO and DRSXHO), in comparison with that occurred in the digestion of the other above-mentioned samples. These results point to an antioxidant effect of this level of ovalbumin during digestion, in agreement both with previous studies carried out with other slightly oxidized oils [37] and with the proven antioxidant ability of ovalbumin and its hydrolysates [66].

As a consequence of the antioxidant effect of this level of ovalbumin, it is evident that a lower concentration of toxic oil oxidation derived compounds able to be absorbed will be formed during digestion compared to those generated in the digestion of the other samples.

### 3.4. Concentration of oil Oxidation Derivatives in the Digestates versus That of the Samples Submitted to Digestion

The samples submitted to digestion contain a broad range of oil oxidation derivatives, as has been described in Section 3.1. In addition, during digestion an additional amount of some oxidation compounds is generated, as a consequence of some degradation of the most unsaturated acyl groups, as proved in Section 3.3. All these oil oxidation derived products could be able to react with digestive enzymes, with other components of the digestive juices or with the added protein, or to evolve under the in vitro digestion conditions. In order to have an approximate idea of their fate during in vitro digestion, their concentrations in the different digestates, either in the absence or in the presence of both proportions of ovalbumin, and also in the oil samples before their being submitted to digestion were studied by ^1^H NMR. Figure 2 and Figure 3a show some of the main ^1^H NMR spectral signals of the oil oxidation derivatives tentatively identified in the different samples subject of study, and Table 3 gives their estimated concentrations, in mmol/mol AG + FA.

#### 3.4.1. Concentrations of Oxidation Derived Compounds in DVSX and DRSX Samples

Most of the oxidation compounds detected in the digestates of VSX and RSX were present in the samples before being submitted to digestion, as Table 3 shows. A detailed analysis of the most important changes observed in the various digestates with regard to the occurrence and concentration of oxidation products in the samples before digestion is made below.

**Figure 5:**
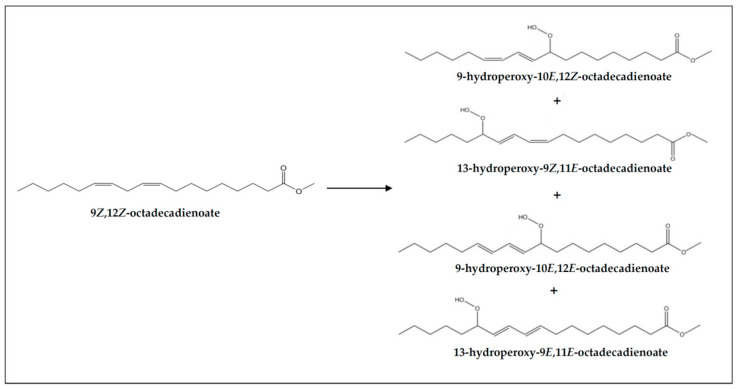
Monohydroperoxy-(*Z*,*E*)- and -(*E*,*E*)-conjugated octadecadienoates formed in the oxidation of 9*Z*,12*Z*-octadecadienoate groups (linoleic acyl groups).

**Figure 6:**
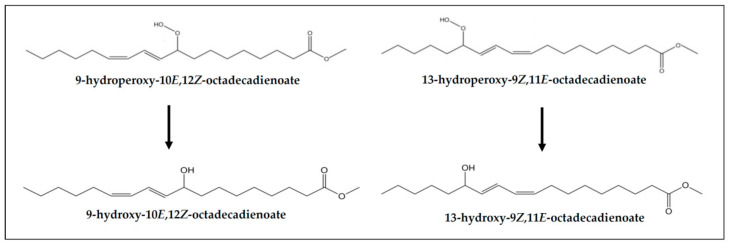
Monohydroxy-(*Z*,*E*)-conjugated dienes and their precursors monohydroperoxy-(*Z*,*E*)-conjugated dienes derived from 9*Z*,12*Z*-octadecadienoate groups (linoleic acyl groups).

**Figure 7:**
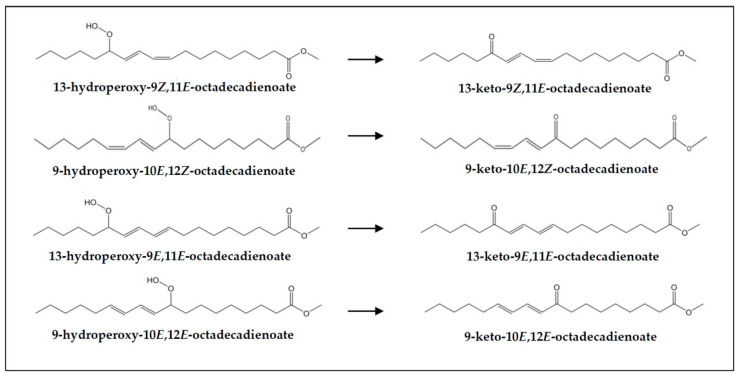
Keto-(*Z*,*E*)- and -(*E*,*E*)-conjugated octadecadienoates derived from their corresponding precursors monohydroperoxy-(*Z*,*E*)- and -(*E*,*E*)-conjugated octadecadienoates.

(a) *Concerning monohydroperoxy-conjugated dienes*. Both monohydroperoxy-(*Z*,*E*)- and -(*E*,*E*)-conjugated dienes are present in the samples before digestion and also in their digestates (see signals “a” and “b” in Figure 2, and estimated concentrations in Table 3). However, a decrease in their concentrations, especially in the case of the (*E*,*E*)-isomers, takes place during digestion, in accordance with that observed previously in slightly oxidized soybean oil samples [9]. This means that part of the monohydroperoxides present in the non-digested samples evolve during in vitro digestion to give other new compounds, and that even potential reactions with proteins or with other compounds present in the digestive juices could have taken place [67]. However, these findings do not indicate that hydroperoxide generation has not occurred during digestion, but that their rate of transformation has been higher than that of their formation. Figure 5 shows the monohydroperoxy-(*Z*,*E*)- and -(*E*,*E*)-conjugated dienes derived from 9*Z*,12*Z*-octadecadienoate groups (linoleic acyl groups) oxidation. The mechanism of formation of these compounds through free radicals is well known [68].

(b) *Concerning dihydroperoxy dienes*. The dihydroperoxides present in VSX and RSX samples disappear during digestion (see signal “c” in Figure 2). These types of compounds have been described as intermediates in the formation of other oxidation derived compounds such as aldehydes [69,70], and their disappearance could be due to their role as intermediate oxidation products. Their formation mechanism is detailed in several papers [69,70].

(c) *Concerning hydroperoxy-epoxy-(E)-monoenes*. The concentration of this kind of compounds (see signal “d1” in Figure 2 and estimated concentrations in Table 3) is smaller in the digestates than in the corresponding undigested samples. This could be due, as commented in the case of monohydroperoxides, to their reaction either with protein amino acid residues or with other digestive juice components, since this class of compounds bear two reactive groups (hydroperoxy and epoxy), and/or to their evolution to bring about other compounds. In this regard, it is worth noticing that hydroperoxy-epoxy-(*E*)-monoenes could be intermediates in the formation of keto-epoxy-derivatives [71].

(d) *Concerning monohydroxy-conjugated dienes*. This class of secondary oxidation products were not present in the samples before digestion (VSX and RSX). However, monohydroxy-(*Z*,*E*)-conjugated dienes are present in their digestates (see signal “z” in Figure 2, signal assignment in Table S9 and estimated concentrations in Table 3), indicating that they are formed during digestion. Although several mechanistic pathways have been proposed for their generation in oxidative processes, their presence in the digestates could be attributed to the reduction of part of the hydroperoxides present in the samples before digestion [72] (see Figure 6). Despite only monohydroxy-(*Z*,*E*)-dienes being detected in the digestates of VSX and RSX samples, the formation of monohydroxy-(*E*,*E*)-conjugated dienes during digestion cannot be discarded, because their ^1^H NMR signals could overlap with others. In fact, the generation of both types of monohydroxy-diene-derivatives (*Z*,*E* and *E*,*E*) has been observed in the in vitro digestion of other slightly oxidized oils [9,15]. These results are in line with the generation of linoleic acid hydroxides from linoleic acid hydroperoxides in the stomach of rats reported by Kanazawa and Ashida [31].

(e) *Concerning keto-conjugated dienes*. As commented in Section 3.1, keto-(*Z*,*E*)- and -(*E*,*E*)-conjugated dienes are present in both undigested samples (VSX and RSX) and digestates (DVSX and DRSX), their concentration being higher in the latter than in the former, probably due to their generation from the respective monohydroperoxy-(*Z*,*E*)- and -(*E*,*E*)-diene precursors (see signals “f” and “e” in Figure 2 and estimated concentrations in Table 3). Figure 7 shows the keto-(*Z*,*E*)- and -(*E*,*E*)-conjugated octadecadienoates derived from their corresponding precursors monohydroperoxy-(*Z*,*E*)- and -(*E*,*E*)-conjugated dienes. For the formation mechanism see reference [73].

(f) *Concerning aldehydes*. As can be observed in Table 3 and Figure 3a, most of the aldehydes found in the digestates DVSX and DRSX are the same as those detected before in the undigested samples VSX and RSX. Exceptions are (*Z*)-2-alkenals and 4-oxo-(*E*)-2-alkenals, which were only detected in the undigested samples (signals “o” and “n” in Figure 3a).

It is worth noting the potential formation during digestion of compounds having both carboxylic and aldehyde groups. They are not indicated in Table 3 as a differentiated class of aldehydes, and are included in the group of n-alkanals. The occurrence of this kind of compounds in DVSX and DRSX is deduced by the appearance in their ^1^H NMR spectra of signals attributable to them. Thus, as Figure 3a shows, the triplet signal “m” of the aldehydic proton of saturated aldehydes present in the spectra of undigested samples VSX and RSX, appears to overlap with that of the aldehydic proton of saturated acid-aldehydes “m1” in the digested samples (signal assignment in Table S6). In addition, as Figure 3a also shows, in the ^1^H NMR spectra of the undigested samples VSX and RSX two signals appear between 2.40 and 2.43 ppm, attributable to different methylenic protons present in these oxidized oils. However, in the spectra of the lipids of their digestates these signals appear to overlap with the, in their turn, overlapped double triplets of n-alkanals and of n-oxo-acids, shown in Figure 3b. The assignment of the signals of the saturated acid-aldehydes is in agreement with ^1^H NMR spectral data of the standard compound 9-oxononanoic acid and with published data [74].

The formation of 9-oxononanoic acid has been described in the digestion by rats of trilinoleoylglycerol hydroperoxides [31]. Furthermore, the formation of acid aldehydes in the in vitro digestion of oxidized oils, as in this case, should be expected. This is because the oxidized oils can contain truncated acyl groups supporting aldehyde groups, whose hydrolysis during digestion gives rise to the formation of acid aldehydes. In addition, 9-oxononanoic acid is one of the main compounds expected to be formed, because the major acyl group in soybean oil is linoleic.

The aldehydes that are in higher concentration in digestates DVSX and DRSX than in the undigested samples VSX and RSX are n-alkanals and 2,3-epoxyaldehydes (see Table 3). According to Onyango [75], these latter (signal “g” in Figure 3a) could derive from the epoxidation of 2-alkenals. The more elevated concentration of n-alkanals (signal “m” in Figure 3a in the digestates than in the undigested samples could be attributed mainly to their low reactivity and, as a consequence, to their greater tendency to accumulate than that of the rest of aldehydes. A higher concentration of n-alkanals in digested than in non-digested samples has also been observed in the study of the in vitro digestion of slightly oxidized samples both of linseed oil [10] and of virgin and refined soybean oils [9]. 

Most of the rest of aldehydes, namely, 4-hydroperoxy- + 4-hydroxy-(*E*)-2-alkenals, 4,5-epoxy-(*E*)-2-alkenals, (*E*)-2-alkenals, (*Z*)-2-alkenals and 4-oxo-(*E*)-2-alkenals (signals “l+k”, “j”, “h”, “o” and “n” in Figure 3a, and estimated concentrations in Table 3), are present in higher levels in the undigested VSX and RSX samples than in their corresponding digestates DVSX and DRSX. This concentration diminution occurring during in vitro digestion, which is very pronounced for 4-hydroperoxy- + 4-hydroxy-(*E*)-2-alkenals, could be due to reactions between the carbonyl group of aldehydes with enzymes (for example through the well-known Maillard reaction) or with other digestive juice components [76,77,78,79]. It is also observed that those aldehydes being in the smallest concentrations in the undigested samples such as (*Z*)-2-alkenals and 4-oxo-(*E*)-2-alkenals, cannot be detected in their digestates for the reasons mentioned above.

**Figure 8:**
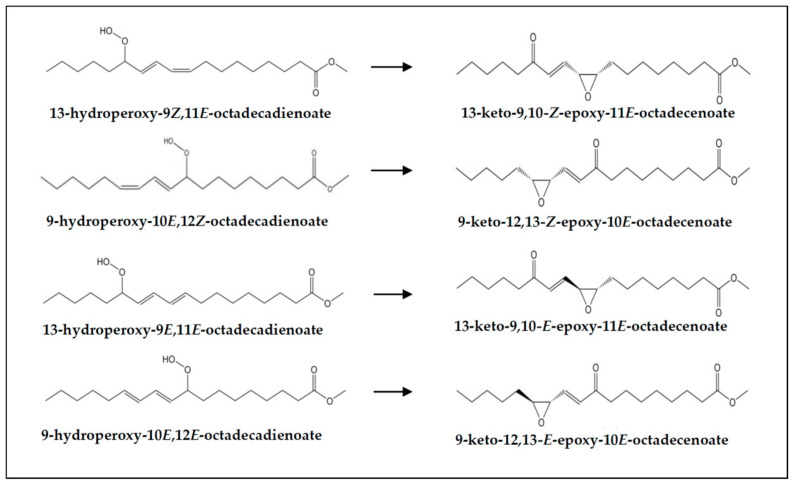
Non-vicinal keto-(*Z*)-epoxy- and keto-(*E*)-epoxy-(*E*)-octadecenoates and their potential monohydroperoxy-(Z,*E*)- and -(*E*,*E*)-octadecadienoate precursors.

(g) *Concerning monoepoxides*. With respect to (*Z*)-monoepoxides, Table 3 shows that their concentration hardly varies during digestion (see samples DVSX and DRSX in comparison with VSX and RSX). As far as (*E*)-monoepoxides are concerned, the signal given by this type of compounds could not be properly identified in the digestates of samples VSX and RSX (see Figure 2, signal “q”), so they were not quantified.

(h) *Concerning formic acid and formate groups*. Formic acid, which was present in VSX and RSX samples (signal “r” in Figure 2 and estimated concentrations in Table 3), was not detected in the lipid extracts of the digestates, possibly because it is retained in their aqueous phase. The concentration of formate groups, in turn, does not exhibit significant changes after digestion (signal “s” in Figure 2 and estimated concentrations in Table 3), this indicating that this type of ester group is not affected by digestive lipolytic enzymes.

(i) *Concerning structures with 5-alkyl-(5H)-furan-2-one group*. Table 3 reveals that this class of furan-derivatives is in higher concentration in DVSX and DRSX than in VSX and RSX samples (see also signal “t” in Figure 2), indicating an advance of the oxidation process during in vitro digestion. As far as we know, this is the first time that the generation of this type of oxidation derived structures during the in vitro digestion of oils has been evidenced by ^1^H NMR. However, this class of furan-derivatives have already been detected in the volatile fraction of other types of non-oxidized in vitro digested oils studied previously [13,38].

(j) *Concerning compounds tentatively identified as hydroxy- or ether-derivatives*. The concentration of the compounds tentatively identified as dihydroxy-derivatives in VSX and RSX samples (giving signal “u”) does not significantly vary after digestion (see Table 3). These outcomes contrast to some extent with that posed by other authors, according to whom diols could be generated from epoxy groups under acidic conditions in gastric medium [80]. Regarding the other type of tentative hydroxy- or ether-derivatives detected in VSX and RSX samples, giving signal “v”, it was not possible to estimate their concentration after digestion, since this signal overlaps with that of 1-MG, much more abundant in the digestates (see Figure 2 and signal “I” in Figure 1 and Table S2).

(k) *Concerning keto-epoxy-(E)-monoenes*. As before mentioned (see Section 3.1), non-vicinal keto-(*Z*)-epoxy- and keto-(*E*)-epoxy-(*E*)-monoenes are present in VSX and RSX samples, and also in their digestates, in these latter in higher concentration than in the former (see signals “x”, “w1” and “w2” in Figure 2, and estimated concentrations in Table 3). These kinds of compounds could proceed directly from monohydroperoxy-dienes (see Figure 8), and indeed Kanazawa and Ashida [31] described their generation from the decomposition of linoleic acid hydroperoxides in the stomach of rats. Notwithstanding, they could also come from keto-dienes oxidation or from hydroperoxy-epoxy-(*E*)-monoenes, as suggested above. See the formation mechanism in reference [73].

By contrast, vicinal keto-(*E*)-epoxy-(*E*)-monoenes, which were present in very low concentrations in VSX and RSX samples (signal “y” in Figure 2), disappear during digestion. This could be due to the substantially higher reactivity towards protein residues described for this group of keto-epoxy-compounds in comparison with those exhibiting non-vicinal keto- and epoxy-groups [74], which might have led to their reaction with some of the proteins present in the digestive fluids.

In summary, different trends in the evolution of the concentration of the various types of oxidation products during digestion have been found. Thus, in general terms, the levels of monohydroperoxy-conjugated-diene-, dihydroperoxy-non-conjugated-diene- and hydroperoxy-epoxy-(*E*)-monoene-derivatives diminish during in vitro digestion, probably due to their role as precursors or intermediates in the generation of other derived oxidation products; notwithstanding, a potential reaction with components of the digestive juices should not be discarded either. Possibly as a consequence of the evolution suffered by some of these oxidation products supporting hydroperoxy groups, the concentrations of others bearing keto groups such as keto-conjugated-diene- and non-vicinal keto-(*E*)-epoxy-(*E*)-monoene-derivatives rise during digestion. Furthermore, it is also noteworthy that an increase in the concentration of other oxidation products, such as saturated aldehydes and compounds having 5-alkyl-(5H)-furan-2-one structure, occurs during digestion. Conversely, other types of more reactive oxidation products, such as unsaturated aldehydes, especially the oxygenated α,β-unsaturated ones, and vicinal keto-(*E*)-epoxy-(*E*)-monoenes decrease, or even disappear, during digestion, this being attributable to their reaction with proteins or with other components present in the digestive juices. In addition, some types of oxidation products not present in the undigested oils, such as monohydroxy-conjugated-diene-derivatives are formed during digestion. Finally, the levels of (*Z*)-monoepoxides and of some tentatively identified dihydroxy-derivatives, as well as of compounds having formate group, hardly vary during the digestion process.

#### 3.4.2. Concentrations of Oxidation Derived Compounds in the Digestates of Samples Containing Ovalbumin Added, DVSXLO, DRSXLO, DVSXHO and DRSXHO

The oxidation compounds detected in the digestates of the oil samples containing ovalbumin are the same as those found in the digestates of samples VSX and RSX, although differences are observed in their concentrations depending on the protein amount.

In the digestates of oil samples having a low ovalbumin proportion, DVSXLO and DRSXLO, in general, no significant differences in the concentrations of oxidation products regarding those found in DVSX and DRSX were observed. However, in most cases a trend towards lower values could be intuited, except in the case of monohydroxy-dienes, where the trend is in the opposite direction (see Table 3).

However, in the digestates of the samples having a high proportion of ovalbumin, DVSXHO and DRSXHO, as Table 3 shows, the concentrations of monohydroperoxy-dienes, aldehydes, structures having 5-alkyl-(5H)-furan-2-one groups, structures having hydroxy-groups different from monohydroxy-conjugated dienes and non-vicinal keto-(*E*)-epoxy-(*E*)-monoenes, are lower than those found in the rest of digested samples. Furthermore, although with statistically not significant differences, lower levels of hydroperoxy-epoxy-(*E*)-monoenes, and keto-dienes were also found in the digestates of the oil samples containing a high proportion of ovalbumin than in the other ones. However, the opposite is true for the concentration of monohydroxy-(*Z*,*E*)-dienes; these probably come from monohydroperoxy-dienes reduction, because the increase in the concentration of the former with digestion is of a similar order to the decreased concentration of the latter, in comparison with the rest of digestates. This transformation could be attributed, among other reasons, to the known reducing power of ovalbumin at this level, which is reinforced by previous findings concerning slightly oxidized oils [9,37].

In brief, the lower concentrations of some oxidation products in the digestates of these highly oxidized oils in presence of a high proportion of ovalbumin could be due to the role of the protein as antioxidant, and to potential reactions between the protein and carbonyl or other functional groups present in oil-derived oxidation compounds. The results here obtained show that the presence of a high ovalbumin proportion during in vitro digestion of this oxidized oil entails a reduction in the concentration of some toxic oxidation products available to react with components of the gastrointestinal tract and/or to be absorbed.

#### 3.4.3. Bioaccessibility of Oxidation Compounds

When it comes to trying to evaluate the bioaccessibility of the oxidation products, both their concentration in the digestates and whether they are absorbable molecules must be taken into account. Their concentration in the digestates has been estimated in the previous section. However, they can be small absorbable molecules or be anchored either on absorbable monoglyceryl monomers or on non-absorbable di- or triglycerides, as well as on dimers, oligomers or more complex polymers. Therefore, both the lipolysis degree reached during digestion transforming tri- and diglycerides into absorbable fatty acids and monoglycerides, and the ability of the juices and digestive conditions to break polymers, oligomers or dimers formed during oil oxidation in absorbable units, will determine the percentage of absorbable oxidation products. However, the information provided by the methodology here used does not permit one to distinguish the absorbable from the non-absorbable oxidation products, for which reason no conclusions about their bioaccessibility can be reached from the available data. Nevertheless, although only some of the oxidation products present in the digestates are expected to be absorbable, all of them will be in contact with digestive tissues.

### 3.5. Some Remarks about the Relevance of the Presence of Oxidation Products in the Gastrointestinal Tract

In the light of the outcomes of this work, it is evident that an important proportion of the oxidation products present in the highly oxidized oils here studied remains after the in vitro digestion process. As already mentioned above, the bioaccessibility of all these compounds will depend on whether they are supported or not on absorbable molecules. Notwithstanding, whether they are absorbed or not, their presence in the gastrointestinal tract could negatively affect human health, since damage of the intestinal barrier due to reactions with oxidation compounds can trigger the development of various pathologies [81]. In this respect, monohydroperoxides have been shown to induce oxidative damage and cell death in the colon, and this has been suggested to contribute to an enhanced risk of colon cancer [62]. In addition, the inhibition of gastrointestinal detoxifying enzymes by their reaction with aldehydes can favor a greater absorption of certain oxidation products [7].

This work also evidences that, of all the various classes of oxidation derivatives studied, epoxy-compounds, which hardly receive any attention in lipid oxidation studies, remain to a great extent after digestion. This finding is in line with the results of Chalvardjian and coworkers [36], who reported that the epoxy group survived the digestive system, and with those of Wilson and coworkers [29], who observed that ^13^C-labelled epoxides were absorbed intact. This can be considered a relevant issue, since among the epoxides present in the oxidized oils here studied, toxic monoepoxides of linoleic groups such as leukotoxin and isoleukotoxin [19,82] could be present. In addition, the increased concentrations of keto-epoxy-(*E*)-monoenes observed after digestion should also be highlighted, since this type of compounds are able to covalently modify proteins and have been related to a great variety of deleterious health effects associated with inflammatory processes and respiratory diseases, among others [21,22]. Therefore, although epoxides are considered in general as very reactive compounds [77], the results of this study reveal that a considerable proportion of them lingers after digestion, and depending on their absorption extent, they would be able to exert deleterious effects beyond the intestine. It is worth noticing that, within the great variety of epoxy-compounds that can be generated in lipid oxidation processes, as far as we know, only certain monoepoxides have been subject of study concerning their occurrence in the diet, and indeed they have been found both in oils subjected to frying and in fresh oils [4,83,84]. In general, the oxygenated α,β-unsaturated aldehydes have been shown to be much more reactive than epoxides, since they exhibit a considerable concentration decrease during digestion. Despite this, part of them stays in the digestates, so they could also be absorbed and reach other targets. These results offer a new perspective both on the potential role of the various classes of oxidation products coming from dietary sources in biological damage, and on the assessment of the health risks derived from them. Thus, although some types of aldehydes are very reactive and toxic compounds [18], a great proportion of them seems to react with components of the digestive fluids; in contrast, epoxy-compounds and other types of oxidation products that have also been attributed toxicity, such as those with keto-diene structure [63,72] or the tentatively identified dihydroxy-derivatives [23,85], remain in the digestates to a large degree or even increase after the digestion process. Moreover, new oxidation compounds associated with some human pathologies or other adverse effects, such as monohydroxy-conjugated-diene-derivatives [61] and 9-oxononanoic acid [86], can be generated during the in vitro digestion of polyunsaturated oxidized oils.

## 4. Conclusions

The high oxidation level of the oils studied affects negatively the lipolysis extent reached during their in vitro digestion process, this being smaller than that of the same unoxidized or slightly oxidized oils. The presence of a small concentration of ovalbumin during digestion hardly affects the lipolysis extent reached. Furthermore, and in contrast to that observed in the digestion of slightly oxidized oils, the presence of a high concentration of ovalbumin does not improve the lipolysis extent in comparison with that reached in presence of a low concentration of ovalbumin, although a certain difference in the lipolysis pattern is observed. Nevertheless, this difference does not entail important variations in the potential bioaccessibility of the oil main components in relation to that of the other samples.

The high oxidation level of the oils submitted to in vitro digestion promotes the occurrence of oxidation reactions during this process, compared with the in vitro digestion of the same unoxidized or slightly oxidized oils. This is proved by the important diminution in the concentration of polyunsaturated acyl groups due to their degradation during the in vitro digestion of these highly oxidized oils compared with the other oil samples above. The presence of a small concentration of ovalbumin during digestion does not provoke a remarkable beneficial effect in this respect. However, the presence of a high concentration of protein greatly hinders the degradation of polyunsaturated acyl groups during the oil in vitro digestion, although it does not totally avoid it, clearly evidencing its antioxidant power. The effect of the protein at this concentration is twofold: on the one hand, it enables that more lipid nutrients remain non-degraded after digestion in comparison with the other digested samples, and on the other, fewer toxic oil oxidation derivatives are formed during digestion.

The in vitro digestion of these highly oxidized oils in the absence or in the presence of a low concentration of ovalbumin gives rise to a digestate that contains almost the same broad range of oxidation derived compounds as the oil submitted to digestion, with certain differences regarding the concentration of some of them. It is worth highlighting the reduction in the concentration both of hydroperoxy derived compounds and of α,β-unsaturated aldehydes, especially of the very reactive and toxic oxygenated ones, with digestion; however, the opposite is true for the levels of monohydroxy- and keto-conjugated dienes, of n-alkanals and of non vicinal-keto-epoxy-(*E*)-monoenes. The decline in the concentration of some of these compounds during digestion can be attributed both to their transformation into other ones and to possible reactions with enzymes and other digestive juices components. A high concentration of ovalbumin during digestion promotes a higher conversion of monohydroperoxy- into monohydroxy-conjugated dienes than in the aforementioned samples, possibly due to the reducing power of the protein at this level. Furthermore, a certain reduction in the concentration of most aldehydes and of some keto-derivatives is also found in these digestates, possibly due to the antioxidant effect of the protein and/or to reactions of this with the oxidation compounds mentioned above. Thus, making a global balance between the concentration of oxidation products present in the digestates of these highly oxidized oils in the absence or in the presence of a low ovalbumin proportion, and in the digestates of the samples digested with a high ovalbumin level, a diminution in the total concentration of oxidation products is noticed, which suggests a certain detoxifying effect of the protein in the latter case. Therefore, the results of this work evidence that the ingestion of food lipids together with some kinds of proteins could contribute to reducing the health risks posed by some oxidation products coming from dietary sources.

Finally, the outcomes of this study could be useful in order to obtain a more global view of which type of oxidation compounds can remain or be generated during digestion and then be available for absorption or for reacting with components of the gastrointestinal tract. This would allow a better assessment of the impact on human health of dietary oxidation products.

## Figures and Tables

**Table 1:** Molar percentages of triglycerides (TG%), diglycerides (1,2-DG% and 1,3-DG%), monoglycerides (2-MG% and 1-MG%) and glycerol (Gol%) in relation to the total number of glyceryl structures present in the oxidized virgin and refined soybean oil samples (VSX and RSX) and in their digestates (DVSX and DRSX), as well as in the digestates of VSX and RSX samples mixed with two different levels of ovalbumin: 26% in weight (DVSXLO and DRSXLO) and 260% in weight (DVSXHO and DRSXHO). Different letters within each column indicate a significant difference among the samples corresponding to the same type of oil (*p* < 0.05).

	TG%	1,2-DG%	1,3-DG%	2-MG%	1-MG%	Gol%
VSX	99.01 ± 0.18 b	0.50 ± 0.00 a	-	-	-	nd
DVSX	35.62 ± 0.60 a	13.23 ± 1.76 b	3.46 ± 1.09 a	19.02 ± 1.35 a	8.25 ± 0.85 b	20.43 ± 2.61 a
DVSXLO	34.93 ± 0.88 a	14.74 ± 0.76 bc	2.78 ± 0.92 a	21.90 ± 2.60 a	7.26 ± 0.20 b	18.39 ± 3.83 a
DVSXHO	30.21 ± 3.60 a	16.58 ± 0.22 c	2.96 ± 0.94 a	28.90 ± 2.59 b	5.59 ± 0.65 a	15.77 ± 0.37 a
						
RSX	99.08 ± 0.08 d	0.90 ± 0.06 a	-	-	-	nd
DRSX	35.02 ± 0.47 b	13.23 ± 1.18 b	2.98 ± 0.29 a	20.92 ± 1.04 a	7.27 ± 1.47 b	20.59 ± 1.50 a
DRSXLO	34.09 ± 0.11 b	14.34 ± 0.55 b	2.51 ± 0.02 a	23.95 ± 1.87 b	7.44 ± 0.22 b	17.68 ± 1.40 a
DRSXHO	31.51 ± 0.49 a	17.01 ± 1.59 c	3.22 ± 1.35 a	27.16 ± 0.75 c	4.35 ± 1.16 a	16.74 ± 1.86 a

-: not detected; nd: not determined.

**Table 2:** Concentrations of the two kinds of polyunsaturated acyl groups+fatty acids (AG + FA), expressed in mmol/mol AG + FA, present in the oxidized virgin and refined soybean oil samples (VSX and RSX) and in their digestates (DVSX and DRSX), as well as in the digestates of VSX and RSX samples mixed with two different levels of ovalbumin: 26% in weight (DVSXLO and DRSXLO) and 260% in weight (DVSXHO and DRSXHO). Different letters within each column indicate a significant difference among the samples corresponding to the same type of oil (*p* < 0.05).

	Linolenic	Linoleic
VSX	10.2 ± 0.4 b	174.4 ± 2.3 c
DVSX	7.0 ± 1.0 a	148.4 ± 9.5 a
DVSXLO	7.1 ± 0.6 a	147.8 ± 2.9 a
DVSXHO	7.6 ± 0.6 a	161.7 ± 3.6 b
		
RSX	7.1 ± 0.5 b	167.7 ± 5.9 b
DRSX	3.9 ± 1.1 a	134.1 ± 9.3 a
DRSXLO	3.5 ± 0.4 a	134.8 ± 3.6 a
DRSXHO	6.9 ± 0.7 b	159.1 ± 7.5 b

**Table 3:** Concentrations of the several kinds of oxidation structures and compounds, expressed in mmol/mol acyl groups+fatty acids, present in the oxidized virgin and refined soybean oil samples (VSX and RSX) and in their digestates (DVSX and DRSX), as well as in the digestates of VSX and RSX samples mixed with two different levels of ovalbumin: 26% in weight (DVSXLO and DRSXLO) and 260% in weight (DVSXHO and DRSXHO). Different letters within each row indicate a significant difference among the samples corresponding to the same type of oil (*p* < 0.05). 4-OOH: 4-hydroperoxy; 4-OH: 4-hydroxy.

	Samples Derived from Virgin Soybean Oil				Samples Derived from Refined Soybean Oil			
	VSX	DVSX	DVSXLO	DVSXHO	RSX	DRSX	DRSXLO	DRSXHO
**Monohydroperoxy-conjugated dienes**								
Hydroperoxy-(*Z*,*E*)-dienes	15.24 ± 0.46 c	8.83 ± 1.04 b	8.32 ± 0.67 b	6.90 ± 0.78 a	11.98 ± 1.99 b	9.07 ± 0.95 a	8.88 ± 1.76 a	7.46 ± 0.71 a
Hydroperoxy-(*E*,*E*)-dienes	23.84 ± 0.64 b	12.53 ± 1.32 a	12.15 ± 2.66 a	9.74 ± 0.87 a	24.44 ± 1.61 b	12.09 ± 0.93 a	11.29 ± 1.12 a	11.03 ± 1.31 a
Total hydroperoxy-dienes	39.08 ± 1.10 c	21.36 ± 2.36 b	20.47 ± 3.33 b	16.64 ± 1.66 a	36.42 ± 3.59 b	21.15 ± 1.89 a	20.18 ± 2.87 a	18.49 ± 2.02 a
**Dihydroperoxy-non-conjugated dienes**	5.12 ± 0.23	-	-	-	5.25 ± 0.51	-	-	-
**Hydroperoxy-epoxy-(*E*)-monoenes**	9.19 ± 0.34 b	5.79 ± 1.08 a	5.58 ± 0.13 a	4.48 ± 0.94 a	14.20 ± 2.04 b	6.82 ± 1.01 a	6.79 ± 0.61 a	5.13 ± 0.43 a
**Monohydroxy-conjugated dienes**								
Hydroxy-(*Z*,*E*)-dienes	-	2.35 ± 0.38 a	2.42 ± 0.34 a	5.03 ± 1.09 b	-	2.04 ± 0.40 a	2.47 ± 0.31 a	4.55 ± 0.66 b
Hydroxy-(*E*,*E*)-dienes	-	nd	nd	nd	-	nd	nd	nd
**Keto-conjugated dienes**								
Keto-(*Z*,*E*)-dienes	2.02 ± 0.19 a	3.79 ± 0.57 b	3.55 ± 0.46 b	2.98 ± 0.55 b	1.63 ± 0.10 a	3.31 ± 0.40 b	3.20 ± 0.25 b	2.80 ± 0.54 b
Keto-(*E*,*E*)-dienes	3.97 ± 0.13 a	5.42 ± 0.72 b	5.15 ± 0.45 b	4.81 ± 0.24 b	4.31 ± 0.28 a	5.68 ± 1.02 a	5.41 ± 0.86 a	4.95 ± 0.66 a
**Aldehydes**								
n-Alkanals	1.59 ± 0.16 a	4.27 ± 0.53 c	3.75 ± 0.72 bc	3.15 ± 0.29 b	2.25 ± 0.15 a	4.71 ± 0.87 b	4.50 ± 1.10 b	3.55 ± 0.51 ab
4-OOH-+4-OH-(*E*)-2-alkenals	7.48 ± 0.30 c	2.24 ± 0.34 b	1.78 ± 0.16 b	1.24 ± 0.18 a	9.01 ± 0.58 b	2.25 ± 0.14 a	2.16 ± 0.64 a	1.76 ± 0.30 a
4,5-Epoxy-(*E*)-2-alkenals	0.62 ± 0.06 b	0.42 ± 0.10 a	0.34 ± 0.10 a	-	0.87 ± 0.23 b	0.38 ± 0.17 a	0.34 ± 0.16 a	-
(*E*,*E*)-2,4-Alkadienals	0.91 ± 0.11 b	0.90 ± 0.10 b	0.92 ± 0.02 b	0.64 ± 0.11 a	0.99 ± 0.18 a	0.91 ± 0.19 a	0.89 ± 0.14 a	0.70 ± 0.15 a
(*E*)-2-Alkenals	3.23 ± 0.21 d	2.63 ± 0.53 c	1.79 ± 0.20 b	0.80 ± 0.16 a	3.95 ± 0.19 d	2.36 ± 0.30 c	1.77 ± 0.24 b	1.11 ± 0.18 a
(*Z*)-2-Alkenals	0.64 ± 0.28	-	-	-	0.47 ± 0.11	-	-	-
4-Oxo-(*E*)-2-alkenals	0.16 ± 0.06	-	-	-	0.22 ± 0.05	-	-	-
2,3-Epoxy-alkanals	-	0.35± 0.03 a	0.31 ± 0.03 a	0.29 ± 0.04 a	0.22 ± 0.07 a	0.39 ± 0.10 a	0.39 ± 0.07 a	0.31 ± 0.04 a
**Monoepoxides**								
(*Z*)-Monoepoxides	11.52 ± 1.21 a	11.22 ± 0.61 a	10.74 ± 0.57 a	11.79 ± 0.59 a	14.22 ± 0.42 a	13.40 ± 0.74 a	13.16 ± 1.28 a	12.67 ± 0.83 a
(*E*)-Monoepoxides	2.14 ± 0.02	nd	nd	nd	2.89 ± 0.16	nd	nd	nd
**Formic acid**	0.67 ± 0.07	APS	APS	APS	0.68 ± 0.02	APS	APS	APS
**Formate groups**	3.73 ± 0.48 a	3.61 ± 0.35 a	3.63 ± 0.44 a	4.00 ± 0.38 a	4.00 ± 0.83 a	4.01 ± 0.34 a	3.97 ± 0.27 a	3.82 ± 0.67 a
**5-Alkyl-(5H)-furan-2-one derivatives**	1.36 ± 0.01 a	1.90 ± 0.43 b	1.68 ± 0.37 ab	1.50 ± 0.14 a	1.49 ± 0.15 a	2.67 ± 0.06 c	2.24 ± 0.24 b	1.71 ± 0.13 a
**Other potential hydroxy- or ether-derivatives**								
Giving signal “u” (3.43 ppm)	2.65 ± 0.17 b	2.36± 0.32 ab	2.27 ± 0.27 ab	1.89 ± 0.27 a	3.25 ± 0.27 b	3.16 ± 0.59 b	3.10 ± 0.14 b	1.99 ± 0.39 a
Giving signal “v” (3.62 ppm)	1.85 ± 0.12	nd	nd	nd	2.13 ± 0.19	nd	nd	nd
**Keto-epoxy-(*E*)-monoenes**								
Non-vicinal keto-epoxy-(*E*)-monoenes	1.34 ± 0.04 a	2.68 ± 0.47 b	2.81 ± 0.30 b	2.54 ± 0.15 b	1.66 ± 0.09 a	3.56 ± 0.43 c	3.50 ± 0.21 c	2.85 ± 0.21 b
Non-vicinal keto-(*E*)-epoxy-(*E*)-monoenes nd		1.98 ± 0.49 b	2.03 ± 0.22 b	1.58 ± 0.19 ab	nd	2.64 ± 0.43 b	2.51 ± 0.19 b	1.89 ± 0.18 a
Non-vicinal keto-(*Z*)-epoxy-(*E*)-monoenes nd		0.70 ± 0.04 a	0.78 ± 0.13 a	0.96 ± 0.09 b	nd	0.92 ± 0.17 a	0.99 ± 0.06 a	0.96 ± 0.11 a
Vicinal keto-(*E*)-epoxy-(*E*)-monoenes	0.29 ± 0.04	-	-	-	0.28 ± 0.05	-	-	-

-: not detected; nd: not determined; APS: probably only present in the aqueous phase of the digestate.

## Data Availability

Data sharing not applicable.

## Supplementary Materials

The following are available online at https://www.mdpi.com/article/10.3390/foods10071573/s1, Table S1: Concentration (units given in brackets) of each one of the components present in the juices used in the in vitro digestion procedure, together with the pH of these latter, Table S2: Chemical shift assignments and multiplicities of the ^1^H NMR signals in CDCl_3_ of the main protons of glycerides present in the samples before and/or after in vitro digestion. MG: monoglycerides; DG: diglycerides; TG: triglycerides, Table S3: Chemical shift assignments and multiplicities of the ^1^H NMR signals in CDCl_3_ of the main protons of acyl groups and fatty acids present in the samples before and/or after in vitro digestion. TG: triglycerides; DG: diglycerides; MG: monoglycerides, Table S4: Chemical shifts, multiplicities and assignments of the ^1^H NMR signals, obtained in CDCl_3_, of protons of some hydroperoxy derivatives present in the samples before and/or after in vitro digestion, Table S5: Chemical shifts, multiplicities and assignments of the ^1^H NMR signals, obtained in CDCl_3_, of protons of different types of keto-conjugated dienes present in the samples before and after in vitro digestion, Table S6: Chemical shifts, multiplicities and assignments of the ^1^H NMR signals, obtained in CDCl_3_, of protons of different types of aldehydes present in the samples before and/or after in vitro digestion, Table S7: Chemical shifts, multiplicities and assignments of the ^1^H NMR signals, obtained in CDCl_3_, of protons of different types of oxidation products present in the samples before and/or after in vitro digestion, Table S8: Chemical shifts, multiplicities and assignments of the ^1^H NMR signals, obtained in CDCl_3_, of protons of different types of keto-epoxy-(*E*)-monoene-derivatives present in the samples before and/or after in vitro digestion, Table S9: Chemical shifts, multiplicities and assignments of the ^1^H NMR signals, obtained in CDCl_3_, of protons of different types of some monohydroxy-conjugated-diene derivatives present in the samples after in vitro digestion, Figure S1: Enlargement of some spectral regions of the ^1^H NMR spectra of the lipid extracts of: the digested juices subjected to digestion conditions (DJ); the digestive juices subjected to digestion conditions mixed with ovalbumin at the highest proportion tested (DJHO); the lipid extract of the digestate of sample RSXHO. The signal letters agree with those in Tables S2, S3 and S7. Signals marked with an asterisk are considered to come from the ovalbumin sample used.
