# Supplementary material for: 1H NMR Study of the In Vitro Digestion of Highly Oxidized Soybean Oil and the Effect of the Presence of Ovalbumin
Source: Foods. 2021 Jul 6;10(7):1573. doi: 10.3390/foods10071573 (PMC8307026; doi:10.3390/foods10071573)
Supplement: Supplementary file 1 [file foods-10-01573-s001.zip › Foods-1258425-Supplementary Material-final.pdf]

foods MDPI

SUPPLEMENTARY MATERIAL

^{1}H NMR study of the *in vitro* digestion of highly oxidized soybean oil and the effect of the presence of ovalbumin

Ana S. Martin-Rubio; Patricia Sopelana; María L. Ibargoitia; María D. Guillén*

Food Technology, Faculty of Pharmacy, Lascaray Research Center, University of the Basque Country (UPV/EHU). Paseo de la Universidad nº 7, 01006 Vitoria, Spain.
* Correspondence: Tel: 34-945-013081, Fax: 34-945-013014

Table S1. Concentration (units given in brackets) of each one of the components present in the juices used in the *in vitro* digestion procedure, together with the pH of these latter.

Components	Saliva	Gastric juice	Duodenal juice	Bile juice
KCl (mmol/L)	12.02	11.06	7.57	5.05
NaCl (mmol/L)	5.10	47.09	119.98	89.99
$NaHCO_3$ (mmol/L)	20.17	-	40.33	68.86
NaH_2PO_4 (mmol/L)	7.40	0.22	-	-
NH_4Cl (mmol/L)	-	5.72	-	-
KH_2PO_4 (mmol/L)	-	-	0.59	-
Na_2SO_4 (mmol/L)	4.79	-	-	-
KSCN (mmol/L)	2.06	-	-	-
$MgCl_2$ (mmol/L)	-	-	0.53	-
$CaCl_2$*$2H_2O$ (mmol/L)	-	2.72	1.36	1.51
HCl (37%) (mL/L)	-	6.50	0.18	0.15
Urea (mmol/L)	3.33	1.42	1.67	4.16
Glucose (mmol/L)	-	3.61	-	-
Glucuronic acid (mmol/L)	-	0.10	-	-
Uric acid (mmol/L)	0.09	-	-	-
Glucoseamine hydrochloride (mmol/L)	-	1.53	-	-
Bovine serum albumin (g/L)	-	1.00	1.00	1.80
Mucin (g/L)	0.025	3.00	-	-
Aspergillus oryzae α-amylase (g/L)	0.29	-	-	-
Aspergillus niger lipase (U/mL)	-	100	-	-
Pepsin (g/L)	-	2.50	-	-
Pancreatin (g/L)	-	-	9.00	-
Lipase type II from porcine pancreas (g/L)	-	-	1.50	-
Bovine bile extract (g/L)	-	-	-	18.75
**pH**	6.8±0.2	1.6±0.3	8.1±0.2	8.2±0.2

Some details of the in vitro digestion procedure used.

The digestion experiment commenced by adding 6 mL of saliva to each of the oil samples. After 5 min of incubation, 12 mL of gastric juice was added and the mixture was rotated head-over-heels at 40 rpm for 2 h at 37±2 ºC. 1 hour after starting the gastric digestion, pH was set between 2 and 3 with HCl (37%), simulating the gradual acidification of the chyme occurring *in vivo*. After 2 h of gastric digestion, 2 mL of sodium bicarbonate solution (1 M), 12 mL of duodenal juice and 6 mL of bile juice were added. Subsequently,

pH was set between 6 and 7, and the mixture was rotated again at 40 rpm and incubated at 37±2 ºC for 4 h.

Table S2. Chemical shift assignments and multiplicities of the ^{1}H NMR signals in $CDCl_3$ of the main protons of glycerides present in the samples before and/or after *in vitro* digestion. MG: monoglycerides; DG: diglycerides; TG: triglycerides.

Signal	Chemical shift (ppm)	Multiplicity	Type of protons[a]	Structures
			Glyceride structure protons	
**I**	3.65	dddd	$ROCH_2$-CHOH-C$\underline{H}_2$OH	glyceryl group in **1-MG**
**J**	3.73	m*	$ROCH_2$-CH(O*R*′)-C$\underline{H}_2$OH	glyceryl group in **1,2-DG**
**K**	3.84	m*	HOC$\underline{H}_2$-CH(O*R*)-C$\underline{H}_2$OH	glyceryl group in **2-MG**
**L**	3.94	m	$ROCH_2$-C$\underline{H}$OH-CH_2OH	glyceryl group in **1-MG**
**M**	4.05-4.21	m	ROC$\underline{H}_2$-C$\underline{H}$OH-C$\underline{H}_2$OR′	glyceryl group in **1,3-DG**
**N**	4.18	dddd	ROC$\underline{H}_2$-CHOH-CH_2OH	glyceryl group in **1-MG**
**O**	4.22	dddd	ROC$\underline{H}_2$-CH(O*R*′)-C$\underline{H}_2$OR′′	glyceryl group in **TG**
**P**	4.28	dddd	ROC$\underline{H}_2$-CH(O*R*′)-CH_2OH	glyceryl group in **1,2-DG**
**Q**	4.93	m	$HOCH_2$-C$\underline{H}$(O*R*)-CH_2OH	glyceryl group in **2-MG**
**R**	5.08	m	$ROCH_2$-C$\underline{H}$(O*R*′)-CH_2OH	glyceryl group in **1,2-DG**

Abbreviations: m: multiplet; dddd: doublet of double doublets; *This signal shows different multiplicity if the spectrum is acquired from the pure compound or taking part in the mixture. [a]Assignments taken from Nieva-Echevarría, B.; Goicoechea, E.; Manzanos, M.J.; Guillén, M.D. *Food Res. Int.* **2014**, *66*, 379-387.

Table S3. Chemical shift assignments and multiplicities of the ^{1}H NMR signals in $CDCl_3$ of the main protons of acyl groups and fatty acids present in the samples before and/or after *in vitro* digestion. TG: triglycerides; DG: diglycerides; MG: monoglycerides.

Signal	Chemical shift (ppm)	Multiplicity	Type of protons[a,b]	Structures
			Main acyl groups (AG) and fatty acids (FA)	
**A**	0.88	t	-C$\underline{H}_3$	saturated and monounsaturated **ω-9 AG** and **FA**
	0.89	t	-C$\underline{H}_3$	linoleic **AG** and **FA**
**B**	0.97	t	-C$\underline{H}_3$	linolenic **AG** and **FA**
**C**	1.19-1.42	m**	-(C$\underline{H}_2$)$_n$-	**AG** and **FA**
**D**	1.61	m	-OCO-CH_2-C$\underline{H}_2$-	**AG** in **TG**
	1.62	m	-OCO-CH_2-C$\underline{H}_2$-	**AG** in **1,2-DG**
	1.63	m	-OCO-CH_2-C$\underline{H}_2$-, COOH-CH_2-C$\underline{H}_2$-	**AG** in **1,3-DG**, **1-MG** and **FA**
	1.64	m	-OCO-CH_2-C$\underline{H}_2$-	**AG** in **2-MG**
**E**	1.92-2.15	m***	-C$\underline{H}_2$-CH=CH-	**AG** and **FA**
**F**	2.26-2.36	dt	-OCO-C$\underline{H}_2$-	**AG** in **TG**
	2.33	m	-OCO-C$\underline{H}_2$-	**AG** in **1,2-DG**
	2.35	t	-OCO-C$\underline{H}_2$-, COOH-C$\underline{H}_2$-	**AG** in **1,3-DG**, **1-MG** and **FA**
	2.38	t	-OCO-C$\underline{H}_2$-	**AG** in **2-MG**
**G**	2.77	t	=HC-C$\underline{H}_2$-CH=	linoleic **AG** and **FA**
**H**	2.80	t	=HC-C$\underline{H}_2$-CH=	linolenic **AG** and **FA**

Abbreviations: d: doublet; t: triplet; m: multiplet; **Overlapping of multiplets of methylenic protons in the different AG and FA either in β-position, or further, in relation to double bonds, or in γ-position, or further, in relation to the carbonyl group; ***Overlapping of multiplets of the α-methylenic protons in relation to a single double bond of the different unsaturated acyl groups.

[a]Assignments of AG in TG taken from Guillén, M.D.; Ruiz, A. *J. Sci. Food Agric.* **2003**, *83*, 338-346.

[b]Assignments of AG in partial glycerides (DG and MG) and of FA taken from Nieva-Echevarría, B.; Goicoechea, E.; Manzanos, M.J.; Guillén, M.D. *Food Res. Int.* **2014**, *66*, 379-387.

Table S4. Chemical shifts, multiplicities and assignments of the ^{1}H NMR signals, obtained in $CDCl_3$, of protons of some hydroperoxy derivatives present in the samples before and/or after *in vitro* digestion.

Signal	Chemical shift (ppm)	Multiplicity	Functional group	
			Type of protons	Compounds and/or family of compounds
			Monohydroperoxides[a]	
-	8.48	dd	-OOH	9-hydroperoxy-10*E*,12*Z*-octadecadienoate 13-hydroperoxy-9*Z*,11*E*-octadecadienoate**
**a**	***6.55***	dddd	-C**H**=CH-*	
-	5.99	ddtd	-CH=CH-	
-	5.57	ddm	-CH=CH-	
-	5.48	dtm	-CH=CH-	
-	8.42	bs	-OOH	9-hydroperoxy-10*E*,12*E*-octadecadienoate 13-hydroperoxy-9*E*,11*E*-octadecadienoate**
**b**	***6.24***	ddm	-C**H**=CH-	
-	6.03	ddtd	-CH=CH-	
-	5.72	dtm	-CH=CH-	
-	5.47	ddm	-CH=CH-	
	Dihydroperoxides[b]			
**c**	***4.82***	dd	-C**H**-OOH	9,12-dihydroperoxy-10*E*,13*E*-dienes + 10,13-dihydroperoxy-8*E*,11*E*-dienes
-	4.30-4.39	m	-CH-OOH	
	Hydroperoxy-epoxy-monoenes[c]			
**d1**	***5.85***	dd	-C**H**=CH-	9-hydroperoxy-12,13-(*E*)-epoxy-10*E*-octadecenoate
**d2**	3.11	dd	-HCOCH-	

Abbreviations: bs: broad signal; d: doublet; t: triplet; m: multiplet; dd: double doublet; dddd: doublet of double doublets. *Area of the signals due to the protons in bold were used for the quantification of each kind of compounds, using [equation S13]. **The assignment of the ^{1}H NMR signals of the protons was made with the aid of standard compounds.

[a]Assignments taken from Goicoechea, E.; Guillén, M.D. *J. Agric. Food Chem.* **2010**, *58*, 6234-6245.

[b]Assignments taken from Zhang, W. Synthesis and Fragmentation Reactions of Linoleic Acid-Derived Hydroperoxides; Doctoral dissertation, Case Western Reserve University, 2008.

[c]Assignments taken from Gardner, H.W.; Weisleder, D.; Kleiman, R. *Lipids* **1978**, *13*, 246-252.

Table S5. Chemical shifts, multiplicities and assignments of the ^{1}H NMR signals, obtained in $CDCl_3$, of protons of different types of keto-conjugated dienes present in the samples before and after *in vitro* digestion.

Signal	Chemical shift (ppm)	Multiplicity	Functional group	
			Type of protons[a]	Compounds and/or family of compounds
**e**	***7.13***	dm	-C**H**=CH- (C-11)*	(*E*,*E*)-conjugated double bonds associated with ketodienes of linoleic acyl groups**
**f**	***7.49/7.50***	ddd	-CH=C**H**- (C-11)	(*Z*,*E*)-conjugated double bonds associated with ketodienes of linoleic acyl groups**

Abbreviations: d: doublet; m: multiplet. *Area of the signals due to the protons in bold were used for the quantification of each kind of compounds, using [equation S13]. **The assignment of the ^{1}H NMR signals of the protons was made with the aid of standard compounds.

[a]Assignments taken from Dufour, C.; Loonis, M. *Chem. Phys. Lip*ids. **2005**, *138*, 60-68.

Table S6. Chemical shifts, multiplicities and assignments of the ^{1}H NMR signals, obtained in $CDCl_3$, of protons of different types of aldehydes present in the samples before and/or after *in vitro* digestion.

Signal	Chemical shift (ppm)	Multiplicity	Functional group	
			Type of protons	Compounds and/or family of compounds
**g**	***8.90-9.20***[a]	d	-C**H**O*	2,3-epoxy-alkanals
**-**	3.20	m	-HCOCH-	
**-**	3.10	dd	-HCOCH-	
**h**	***9.49***[b]	d	-C**H**O	(*E*)-2-alkenals**

**i**	***9.52***[b]	d	-C**H**O	(*E*,*E*)-2,4-alkadienals**
**j**	***9.55***[b]	d	-C**H**O	4,5-epoxy-(*E*)-2-alkenals**
-	3.33	dd	-HCOC**H**-	
-	2.96	td	-**H**COCH-	
**k**	***9.57***[b]	d	-C**H**O	4-hydroxy-(*E*)-2-alkenals**
**l**	***9.58***[b]	d	-C**H**O	4-hydroperoxy-(*E*)-2-alkenals**
**m**	***9.75***[b]	t	-C**H**O	n-alkanals**
-	2.43	dt	-C**H**$_2$-CHO	
**m1**	***9.76***	t	-C**H**O	9-oxononanoic acid**
**m2**	2.42	dt	-C**H**$_2$-CHO	
**n**	***9.77***	d	-C**H**O	4-oxo-(*E*)-2-alkenals**
**o**	***10.06***[c]	d	-C**H**O	(*Z*)-2-alkenals

Abbreviations: d: doublet; t: triplet; m: multiplet; dd: double doublet. *Area of the signals due to the protons in bold were used for the quantification of each kind of compounds, using [equation S13]. **The assignment of the ^{1}H NMR signals of the protons was made with the aid of standard compounds.

[a]Assignments taken from Daiboun, T.; Elalaoui, M.A.; Thaler-Dao, H.; Chavis, C.; Maury, G. *Biocatalysis* **1993**, *7*, 227-236.

[b]Assignments taken from Goicoechea, E.; Guillén, M.D. *J. Agric. Food Chem*. **2010**, *58*, 6234-6245.

[c]Assignments taken from Moumtaz, S.; Percival, B.C.; Parmar, D.; Grootveld, K.L.; Jansson, P.; Grootveld, M. *Sci. Rep*. **2019**, *9*, 1-21.

Table S7. Chemical shifts, multiplicities and assignments of the ^{1}H NMR signals, obtained in $CDCl_3$, of protons of different types of oxidation products present in the samples before and/or after *in vitro* digestion.

**Signal**	**Chemical shift (ppm)**	**Multiplicity**	**Functional group**	
			**Type of protons**	**Compounds and/or family of compounds**
			(Z)-Monoepoxides[a]	
**p**	***2.98-2.88***	m	-C**H**O**H**C-*	9,10-(*Z*)-epoxy-12*Z*-octadecenoate**<br>12,13-(*Z*)-epoxy-9*Z*-octadecenoate**
-	2.94	m	-C**H**O**H**C-	12,13-(*Z*)-epoxy-9*Z*,15*Z*-octadecadienoate**
			(E)-Monoepoxides[a]	
**q**	***2.73-2.66***	m	-C**H**O**H**C-	9,10-(*E*)-epoxy-12*Z*-octadecenoate<br>12,13-(*E*)-epoxy-9*Z*-octadecenoate**
			Formic acid[b]	
**r**	***8.01***	s	**H**-COOH	formic acid
			Formate groups[c]	
**s**	***8.17-8.03***	m	>HC-O-C**H**=O	formate group
			5-Alkyl-(5H)-furan-2-one structures[d]	
**t**	***7.45***	dd	-CH=C**H**- (ar.C-4)	5-alkyl-(5H)-furan-2-one
			Dihydroxy structures[a]	
**u**	***3.48-3.37***	m	-OHC**H**-C**H**OH-	9,10-dihydroxy-12*Z*-octadecenoate**<br>12,13-dihydroxy-9*Z*-octadecenoate**
			Monohydroxy- or ether-derivatives[e]	
**v**	***3.62***	m	-C**H**$_2$-OH<br>-C**H**OH-<br>-C**H**-O-C**H**-	primary alcohols**<br>secondary alcohols**<br>ether-derivatives

Abbreviations: s: singlet; m: multiplet; dd: double doublet. *Area of the signals due to the protons in bold were used for the quantification of each kind of compounds, using [equation S13]. **The assignment of the ^{1}H NMR signals of the protons was made with the aid of standard compounds.

[a]Assignments taken from Nilewski, C.; Chapelain, C.L.; Wolfrum, S.; Carreira, E.M. *Org. Lett*. **2015**, *17*, 5602-5605.

[b]Assignment taken from Babij, N.R.; McCusker, E.O.; Whiteker, G.T.; Canturk, B.; Choy, N.; Creemer, L.C.; De Amicis, C.V.; Hewlett, N.M.; Johnson, P.L.; Knobelsdorf, J.A.; Li, F; Lorsbach, B.A.; Nugent, B.M.; Ryan, S.J.; Smith, M.R.; Yang, Q. *Org. Process Res. Dev*. **2016**, *20*, 661-667.

[c]Assignment taken from Harry-O'kuru, R.E.; Biresaw, G.; Tisserat, B.; Evangelista, R. *J. Lipids* **2016**, ID 3128604, 12 pages.
[d]Assignment taken from: Bonete, P.; Najera, C. *J. Org. Chem*. **1994**, *59*, 3202-3209. Braukmüller, S.; Brückner, R. *Eur. J. Org. Chem*. **2006**, *2006*, 2110-2118.
[e]Assignments taken from: Caillol, S.; Desroches, M.; Boutevin, G.; Loubat, C.; Auvergne, R.; Boutevin, B. *Eur. J. Lipid Sci. Technol*. **2012**, *114*, 1447-1459. De Souza, V.H.R.; Silva, S.A.; Ramos, L.P.; Zawadzki, S.F. *J. Am. Oil Chem Soc*. **2012**, *89*, 1723-1731. Lligadas, G.; Ronda, J.C.; Galia, M.; Biermann, U.; Metzger, J.O. *J. Polym. Sci. A Polym. Chem*. **2006**, *44*, 634-645.

Table S8. Chemical shifts, multiplicities and assignments of the ^{1}H NMR signals, obtained in $CDCl_3$, of protons of different types of keto-epoxy-(*E*)-monoene-derivatives present in the samples before and/or after *in vitro* digestion.

Signal	Chemical shift (ppm)	Multiplicity	Functional group	
			Type of protons[a]	Compounds and/or family of compounds
-	6.52	dd	-CH=CH-	13-keto-9,10-(*E*)-epoxy-11*E*-octadecenoate / 9-keto-12,13-(*E*)-epoxy-10*E*-octadecenoate**
**w1**	6.38	d	-CH=CH-	
**x**	***3.20***	dd	-HCOC**H**-*	
-	2.89	td	-HCOCH-	
-	2.53	t	$-CH_2-$	
-	6.66	dd	-CH=CH-	13-keto-9,10-(*Z*)-epoxy-11*E*-octadecenoate/ 9-keto-12,13-(*Z*)-epoxy-10*E*-octadecenoate
-	6.40	d	-CH=CH-	
**w2**	***3.52 / 3.53***	ddd/dd	-HCOC**H**-	
**x**	3.20	dd	-HCOCH-	
-	2.55	t	$-CH_2-$	
-	7.02	dt	-CH=CH-	11-keto-12,13-(*E*)-epoxy-9*E*-octadecenoate / 11-keto-9,10-(*E*)-epoxy-12*E*-octadecenoate
-	6.23-6.16	dt	-CH=CH-	
**y**	***3.34-3.28***	d	-HCOC**H**-	
-	3.04-2.98	ddd	-HCOCH-	
-	2.25	t	$-CH_2-$	

Abbreviations: d: doublet; t: triplet; dd: double doublet. *Area of the signals due to the protons in bold were used for the quantification of each kind of compounds, using [equation S13]. **The assignment of the ^{1}H NMR signals of the protons was made with the aid of standard compounds.
[a]Assignments taken from Lin, D.; Zhang, J.; Sayre, L.M. *J. Org. Chem*. **2007**, *72*, 9471-9480.

Table S9. Chemical shifts, multiplicities and assignments of the ^{1}H NMR signals, obtained in $CDCl_3$, of protons of some monohydroxy-conjugated-diene derivatives present in the samples after *in vitro* digestion.

Signal	Chemical shift (ppm)	Multiplicity	Functional group	
			Type of protons[a]	Compounds and/or family of compounds
**z**	***6.47***	dd	-C**H**=CH-*	(*Z,E*)-conjugated double bonds associated with hydroxides
**-**	5.96	t	-CH=CH-	
**-**	5.65	d	-CH=CH-	
**-**	5.43	dt	-CH=CH-	

Abbreviations: d: doublet; t: triplet; dd: double doublet. *Area of the signals due to the protons in bold were used for the quantification of each kind of compounds, using [equation S13].
[a]Assignments taken from Manini, P.; Camera, E.; Picardo, M.; Napolitano, A.; d'Ischia, M. *Chem. Phys. Lipids* **2005**, *134*, 161-171.

Standard compounds for the identification of some of the oxidation products present in the various samples studied.

Hexanal, (*E*)-2-hexenal, (*E*)-2-heptenal, (*E*)-2-decenal, (*E,E*)-2,4-hexadienal, (*E,E*)-2,4-heptadienal, (*E,E*)-2,4-decadienal, 4,5-epoxy-(*E*)-2-decenal, 12,13-(Z)-epoxy-9Z-octadecenoic acid methyl ester (isoleukotoxin methyl ester),

(*E*)-2-penten-1-ol and 1-hexanol, acquired from Sigma-Aldrich; 4-hydroxy-(*E*)-2-nonenal, 4-hydroperoxy-(*E*)-2-nonenal, 4-oxo-(*E*)-2-nonenal, 9,10-(*Z*)-epoxy-12*Z*-octadecenoic acid (leukotoxin), 9,10-dihydroxy-12*Z*-octadecenoic acid (leukotoxin diol), 12,13-dihydroxy-9*Z*-octadecenoic acid (isoleukotoxin diol), 9-keto-12,13-(*E*)-epoxy-10*E*-octadecenoic acid, 9-keto-10*E*,12*E*-octadecadienoic acid, 9-keto-10*E*,12*Z*-octadecadienoic acid, 13-keto-9*Z*,11*E*-octadecadienoic acid, 12R-hydroxy-9*Z*-octadecenoic acid methyl ester (ricinoleic acid methyl ester), trilinolein hydroperoxides and trilinolein hydroxides, purchased from Cayman Chemical (Ann Arbor, MI, USA); 12,13-(*Z*)-epoxy-9*Z*,15*Z*-octadecadienoic acid and 9-oxononanoic acid, acquired from Cymit Quimica (Barcelona, Spain); and 12,13-(*E*)-epoxy-9Z-octadecenoic acid and 9(S)-hydroxy-10*E*,12*E*-octadecadienoic acid (dimorphecolic acid), acquired from Larodan (Malmö, Sweden).

Quantification from ^{1}H NMR spectral data of several compounds present in the starting oil samples and/or in the lipid extracts of the digestates.

A. Lipolytic products

The number of moles (N) of fatty acids and of all the glycerides present in the lipid samples were expressed as follows:

$N_{2\text{-MG}}=Pc*A_K/4$ [Equation S1]
$N_{1\text{-MG}}=Pc*A_L$ [Equation S2]
$N_{1,2\text{-DG}}=Pc*(A_{I+J}-2A_L)/2$ [Equation S3]
$N_{TG}=Pc*(2A_{4.26\text{-}4.38}-A_{I+J}+2A_L)/4$ [Equation S4]
$N_{1,3\text{-DG}}=Pc*(A_{4.04\text{-}4.38}-2A_{4.26\text{-}4.38}-2A_L)/5$ [Equation S5]
$N_{FA}=(Pc*A_F-6N_{TG}-4N_{1,2\text{-DG}}-4N_{1,3\text{-DG}}-2N_{1\text{-MG}}-2N_{2\text{-MG}})/2$ [Equation S6]
$N_{Gol}=(N_{FA}-N_{1,2\text{-DG}}-N_{1,3\text{-DG}}-2N_{2\text{-MG}}-2N_{1\text{-MG}})/3$ [Equation S7]

where Pc is the proportionality existing between the area of the ^{1}H NMR signals and the number of protons that generate them, A_K, A_L, A_{I+J} and A_F are the areas of the corresponding signals indicated in Tables S2 and S3, and $A_{4.26\text{-}4.38}$ and $A_{4.04\text{-}4.38}$ represent the areas of the signals between 4.26 and 4.38 ppm, and between 4.04 and 4.38 ppm, respectively (see Figure S1). Gol: glycerol

Using these equations, the molar percentages of the different kinds of glycerides in relation to the total number of moles of glyceryl structures present (NT_{GS}) were determined as follows:

$NT_{GS}=N_{TG}+N_{1,2\text{-DG}}+N_{1,3\text{-DG}}+N_{2\text{-MG}}+N_{1\text{-MG}}+N_{Gol}$ [Equation S8]
$G\%=100N_G/NT_{GS}$ [Equation S9]

where G is each kind of glyceride (TG, 1,2-DG, 1,3-DG, 2-MG and 1-MG) and N_G the number of moles of each kind of glyceride.

Finally, the molar percentage of glycerol was estimated by means of the following equation:

$Gol\%=100N_{Gol}/NT_{GS}$ [Equation S10]

Equations [S1-S10] were developed and validated in previous studies (Nieva-Echevarría, B.; Goicoechea, E.; Manzanos, M.J.; Guillén, M.D. *Food Res. Int.* **2014**, *66*, 379-387; Nieva-Echevarría, B.; Goicoechea, E.; Manzanos, M.J.; Guillén, M.D. *Food Chem.* **2015**, *179*, 182-190.

B. Molar concentrations of polyunsaturated acyl groups and fatty acids

The concentrations of linolenic acyl groups plus linolenic fatty acids (Ln) and that of linoleic acyl groups plus linoleic fatty acids (L), expressed as millimoles per mole of the total acyl groups plus fatty acids (AG+FA) present in either the starting oils or the lipid extracts of the digested samples, were estimated by using the following equations:

$$[Ln] = [(A_H/4)/(A_F/2)]*1000 \quad \text{[Equation S11]}$$
$$[L] = [(A_G/2)/(A_F/2)]*1000 \quad \text{[Equation S12]}$$

where A_H and A_G are the areas of signals H and G indicated in Table S3. It must be noted that due to partial overlapping of signals H and G, a previous correction of both areas must be undertaken to properly assess the area corresponding to each one of them. For this purpose, trilinolenin and trilinolein were used as references.

Finally, it should be pointed out that signal F is due to methylenic protons bonded to carbon atoms in α-position in relation to carbonyl/carboxyl groups of AG and FA, modified or not, as well as to carbonyl groups of other compounds formed during the oxidation process, such as aldehydes, keto-(*E*)-epoxy-(*E*)-monoenes and keto-dienes. However, despite the high oxidation level of the studied samples, the inclusion in this signal of methylenic protons in α-position in relation to carbonyl groups which are different from that of AG and FA does not affect the calculations before mentioned, in which A_F is included, because the concentration of oxidation products is negligible in relation with that of AG+FA.

C. Oxidation products

The concentration of the several kinds of oxidation products, expressed as millimoles per mole of the sum of AG+FA present, was estimated by using the following equation:

$$[OP] = [(A_{OP}/n)/(A_F/2)]*1000 \quad \text{[Equation S13]}$$

where A_{OP} is the area of the signal selected for the quantification of each oxidation product (OP), shown in Tables S4-S9. It must be pointed out that in some cases it will be necessary to subtract the area corresponding to other compounds that give signals with the same chemical shift than that being determined.

4-Hydroperoxy- and 4-hydroxy-(*E*)-2-alkenals have been quantified together in order to accurately compare their concentrations before and after *in vitro* digestion, because in the digested samples it is very difficult to determine each one of these kinds of aldehydes separately, due to the almost total overlap of their signals (see Figure 3a, letters "l" and "k", respectively).

Particularities of the quantification of some compounds in the samples digested with a 260% in weight of ovalbumin

As shown in Figure S1 for DRSXHO, in the ^{1}H NMR spectra of the extracts obtained from the samples digested with a high proportion of ovalbumin, some signals attributed to this latter overlap with the signals used to estimate both the molar concentration of (Z)-monoepoxides (see signal "p" in Table S7) and the molar percentages of 1,3-DG and TG (see signals "M" and "O", respectively, in Table S2), so their contribution must be subtracted, especially in the case of (Z)-monoepoxides. For this purpose, ovalbumin was

added to the digestive juices after undergoing the digestion process and this mixture was extracted in the same way as the rest of digested samples; the relative areas of the different ovalbumin signals can be determined from the corresponding ^1H NMR spectrum, free of lipids. This enables one to subtract the area of the signals overlapping with those of lipid components in the spectra of the extracts obtained from the digestates taking as a reference the signals that do not overlap with one another (see Figure S1). It is worth noticing that while some signals coming from ovalbumin sample also overlap with those of *bis*-allylic protons (signals "H+G"), their area is very small in relation to that of the latter, and so can be ignored.

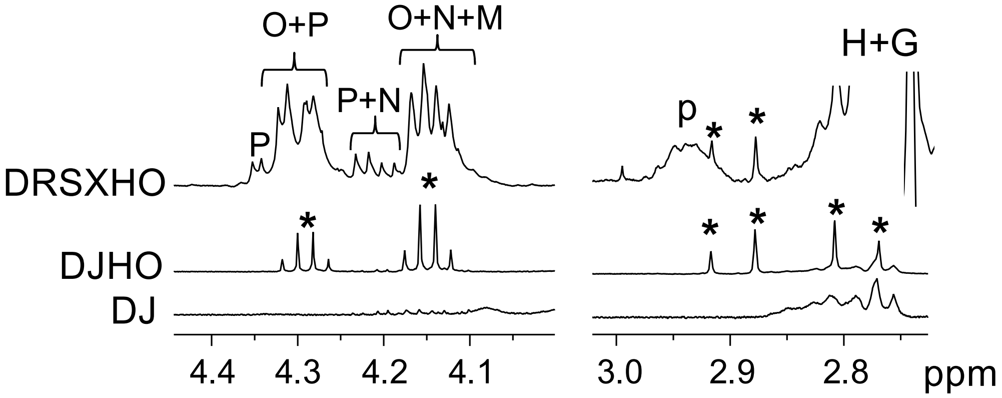

Figure S1. Enlargement of some spectral regions of the ^1H NMR spectra of the lipid extracts of: the digestive juices subjected to digestion conditions (DJ); the digestive juices subjected to digestion conditions mixed with ovalbumin at the highest proportion tested (DJHO); the lipid extract of the digestate of sample RSXHO (DRSXHO). The signal letters agree with those in Tables S2, S3 and S7. Signals marked with an asterisk are considered to come from the ovalbumin sample used.
